# Pathogenicity Factors of Genomic Islands in Intestinal and Extraintestinal *Escherichia coli*

**DOI:** 10.3389/fmicb.2020.02065

**Published:** 2020-09-25

**Authors:** Mickaël Desvaux, Guillaume Dalmasso, Racha Beyrouthy, Nicolas Barnich, Julien Delmas, Richard Bonnet

**Affiliations:** ^1^Université Clermont Auvergne, INRAE, MEDiS, Clermont-Ferrand, France; ^2^UMR Inserm 1071, USC-INRAE 2018, M2iSH, Université Clermont Auvergne, Clermont-Ferrand, France; ^3^Laboratoire de Bactériologie, CHU Clermont-Ferrand, Clermont-Ferrand, France

**Keywords:** *Escherichia coli*, pathogenicity islands, genomic island, virulence, ExPEC, AIEC, InPEC

## Abstract

*Escherichia coli* is a versatile bacterial species that includes both harmless commensal strains and pathogenic strains found in the gastrointestinal tract in humans and warm-blooded animals. The growing amount of DNA sequence information generated in the era of “genomics” has helped to increase our understanding of the factors and mechanisms involved in the diversification of this bacterial species. The pathogenic side of *E. coli* that is afforded through horizontal transfers of genes encoding virulence factors enables this bacterium to become a highly diverse and adapted pathogen that is responsible for intestinal or extraintestinal diseases in humans and animals. Many of the accessory genes acquired by horizontal transfers form syntenic blocks and are recognized as genomic islands (GIs). These genomic regions contribute to the rapid evolution, diversification and adaptation of *E. coli* variants because they are frequently subject to rearrangements, excision and transfer, as well as to further acquisition of additional DNA. Here, we review a subgroup of GIs from *E. coli* termed pathogenicity islands (PAIs), a concept defined in the late 1980s by Jörg Hacker and colleagues in Werner Goebel’s group at the University of Würzburg, Würzburg, Germany. As with other GIs, the PAIs comprise large genomic regions that differ from the rest of the genome by their G + C content, by their typical insertion within transfer RNA genes, and by their harboring of direct repeats (at their ends), integrase determinants, or other mobility loci. The hallmark of PAIs is their contribution to the emergence of virulent bacteria and to the development of intestinal and extraintestinal diseases. This review summarizes the current knowledge on the structure and functional features of PAIs, on PAI-encoded *E. coli* pathogenicity factors and on the role of PAIs in host–pathogen interactions.

## Introduction

*Escherichia coli* is a versatile bacterial species that has an extensive phylogenetic substructure comprising eight phylogroups (A, B1, B2, C, D, E, F, and G) that are roughly linked to the lifestyles of the different strains (Milkman, 1973; Selander et al., 1987; Escobar-Páramo et al., 2004a; Clermont et al., 2019). *E. coli* strains colonize the gastrointestinal tract of human infants and coexist in good health with the host, with strains providing mutual benefits for decades. These commensal strains rarely cause diseases in healthy hosts, as they lack specialized virulence traits; they typically originate from phylogroup A (Herzer et al., 1990; Escobar-Páramo et al., 2004b). However, other *E. coli* strains acquire virulence attributes that confer them with the ability to adapt to new niches and cause intestinal and extraintestinal diseases (Le Gall et al., 2007; Tenaillon et al., 2010). Consequently, *E. coli* strains can be classified into three groups: commensal/probiotic strains, intestinal-pathogenic strains and extraintestinal-pathogenic strains.

Intestinal-pathogenic *E. coli* (InPEC), which are rarely encountered in the fecal flora of healthy hosts, cause gastroenteritis or colitis. Six well-described pathotypes of diarrheagenic *E. coli* (DEC) have been identified: enteropathogenic *E. coli* (EPEC), enterohemorrhagic *E. coli* (EHEC), enterotoxigenic *E. coli* (ETEC), enteroaggregative *E. coli* (EAEC), enteroinvasive *E. coli* (EIEC) and diffusely adherent *E. coli* (DAEC) (Nataro and Kaper, 1998; Kaper et al., 2004). The DEC pathotypes can share certain virulence features; however, each pathotype possesses a specific combination of virulence traits that results in a distinctive pathogenic mechanism. The EPEC strains are not specifically classified as a phylogroup, although some studies preferentially associate them with phylogroup B1 (Reid et al., 2000; Wang et al., 2013). The EHEC are distributed preferentially between phylogroups A and B1 (Askari Badouei et al., 2015; Martins et al., 2015). However, the EHEC of serotype O157: H7 belongs to phylogroup E (Girardeau et al., 2005).

Extraintestinal *E. coli* (ExPEC) are facultative pathogens responsible for 80% of urinary tract infections (UTIs) in outpatients. Infection by ExPEC leads to a large portion of nosocomial UTIs (50%) and is the leading cause of abscesses, accounting for 30% of meningitis in neonates (Gransden et al., 1990; Johnson, 1991; Russo and Johnson, 2000; Foxman, 2003; Nielubowicz and Mobley, 2010). Bacteremia and septic shock can accompany infections at any site. ExPEC typically belong to the phylogroup B2 – an *E. coli* genetic background that accumulates virulence factors – and occasionally to phylogroups D, F or G (Tourret and Denamur, 2016; Clermont et al., 2019). Group B2 has the greatest diversity among all *E. coli* phylogroups (Touchon et al., 2009), suggesting that it has subspecies status and includes subgroups correlated with a flexible gene pool (Le Gall et al., 2007; Lescat et al., 2009). This flexible gene pool comprises genes for various combinations of virulence factors, such as adhesins, iron-acquisition systems, host defense-avoidance mechanisms and toxins (Croxen and Finlay, 2010). The link between the evolutionary lineages of *E. coli*, certain extraintestinal virulence genes and infection sites has led to the concepts of uropathogenic *E. coli* (UPEC), sepsis-associated *E. coli* (SEPEC), neonatal meningitis *E. coli* (NMEC) and avian pathogenic *E. coli* (APEC; which cause extraintestinal infections in poultry). Finally, a non-diarrheagenic InEC pathotype, called adherent-invasive *E. coli* (AIEC), has been noted for its association with inflammatory bowel diseases such as Crohn’s disease (Darfeuille-Michaud et al., 2004). AIEC is considered a pathobiont bacterium rather than a bacterium responsible for acute infection, and it frequently belongs to the phylogroup B2, like ExPEC, and shares with ExPEC the most virulence traits.

Whole-genome *E. coli* phylogeny suggests an evolution of ExPEC from commensal *E. coli* strains that were originally devoid of virulence factors but became pathogenic because of horizontal gene transfers involving transduction, transformation, and conjugation events (Lo et al., 2015). Phages, plasmids and large parts of the genome, designated as genomic islands (GIs), were transferred from one bacterium to another (Benedek and Schubert, 2007; Schubert et al., 2009; Schneider et al., 2011; Messerer et al., 2017). These types of GIs carrying virulence-associated genes were identified in UPEC in the early 1980s by Hacker et al. (1983, 1990) and were designated as pathogenicity islands (PAIs) (Blum et al., 1994). Since then, PAIs have been described from SEPEC, MNEC and diarrheagenic isolates and in other species, such as *Salmonella enterica*. The species *Escherichia coli* and *Salmonella enterica* share 70% of their genome (McClelland et al., 2001) and have recently diverged (Doolittle et al., 1996). The divergence of both species has been, in large part, due the acquisition of specific PAIs and has resulted in very different lifestyles (Nieto et al., 2016). This review summarizes the current knowledge on the structure and functional features of PAIs, on *E. coli* PAI-encoded pathogenicity factors and on the role of PAIs in host-pathogen interactions.

## Structural Features of Pathogenicity Islands

Pathogenicity islands are a group of large (>10 kb) integrative elements that encode one or more virulence genes that are absent from the genomes of non-pathogenic representative bacteria of the same species or of closely related species (Blum et al., 1994; Hacker and Carniel, 2001; Dobrindt et al., 2004; Schmidt and Hensel, 2004). In contrast to other integrative elements, such as bacteriophages, plasmids or integrative and conjugative elements (ICEs), PAIs are non-replicative and lack the ability to self-mobilize.

Comparison of the genomic region of PAIs and the remaining parts of the host genome shows that PAIs have their own genomic characteristics, which is strong evidence of their foreign origin and horizontal acquisition. The G + C contents (i.e., the percentage of guanine and cytosine bases), the frequency of dinucleotides or high-order oligonucleotides and the codon usage in PAIs often differ from those of the host organisms (Groisman and Ochman, 1996; Karlin, 2001; Dobrindt et al., 2004). For instance, the G + C content of the 536 UPEC core genome is 50%, while the G + C content is 41% in the PAIs I_536_, II_536_, IV_536_, and V_536_. In the EPEC genome, the G + C content of the LEE (locus of enterocyte effacement) PAI is only 39%, while the G + C content of the *E. coli* core genome is ∼50%. However, the donor and recipient organisms may possibly have a similar G + C sequence composition, thereby complicating the extraction of PAIs from the core genome. Even for donor and recipient organisms with different sequence compositions, the PAI region can be “ameliorated” throughout evolution, making the sequence composition or codon usage of the PAI region similar to that of the core genome (Lawrence and Ochman, 1997). The divergences in GC content can therefore reflect a recent acquisition and/or an evolutionary mechanism that maintains a divergence in GC content. This divergence can have a functional role, since curved and AT-rich PAIs are preferential targets of the global gene silencer H-NS (Lucchini et al., 2006; Navarre et al., 2006; Oshima et al., 2006). The H-NS-mediated silencing prevents the uncontrolled transcription of genes within PAIs to ensure that bacterial fitness is maintained, and it may also have evolutionary consequences by influencing the acquisition and maintenance of foreign DNA (Lucchini et al., 2006; Navarre et al., 2006; Oshima et al., 2006).

**FIGURE 1:**
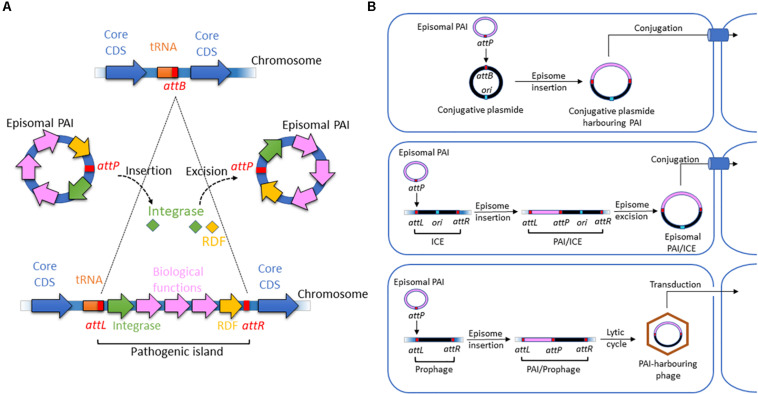
Structure and motility functions of PAIs. **(A)** Insertion and excision processes of PAIs. PAIs are chromosomal fragments of pathogenic bacteria that encode biological functions involved in virulence. Their insertion in the chromosome is due to the presence of *att* sites at a chromosomal acceptor site (*attB*) and in the episomal PAI (*attP*). They are recognized by integrases, which catalyze a recombination of *att* sites. It results in the insertion of the episomal element at the *attB* site and the formation of direct repeated sequences (DRS) also named *attL* (left DRS) and *attR* (right DRS) in the ends of the inserted PAI. The excision of the PAI results from recombination between the direct repeats *attL* and *attR*. Catalyzed by integrases and recombination directionality factors (RDFs) also called excisionases, it generates an episomal element that contains one of the *att* sites (*attP*), while the other *att* site remains in the chromosome (*attB*). **(B)** Horizontal transfer of PAIs via conjugative plasmids, ICEs, and phages harboring *att* sites. Episomal PAIs can be inserted at *att* sites in conjugative plasmids, ICEs and phages as described above and then transferred into a bacterial recipient via conjugation for ICE-type and plasmid-type navettes or via transduction for phage-type navettes.

Nonetheless, most GIs contain a recombination module (Figure 1A) that is also observed in other integrative elements, such as phages, integrons, conjugative transposons and ICEs. The module consists of three parts: (i) an integrase of the tyrosine recombinase family; (ii) two flanking attachment sites forming direct repeats resembling the *att*R and *att*L sites of prophages; and, in some cases, and (iii) a recombination directionality factor (RDF). This recombination module is characteristic of the integrative elements that insert a circular intermediate into the host genome. However, island-encoded integrases form a separate clade within the tyrosine recombinase family (Boyd et al., 2009). Therefore, PAIs are a distinct class of integrative elements and are not degenerate remnants of other mobile elements (Fogg et al., 2011).

Most island-related integrases are inserted adjacent to tRNA or transfer-messenger RNA (tmRNA) loci (Figure 1A). However, the number of specific tRNA genes used is limited. Within the *E. coli* genome, ∼87 tRNA genes have been annotated, and integrative genetic elements use only a handful of these sites. Fifteen tDNAs are hotspots of integration, with *asnT*, *aspV*, *leuX*, *metV*, *pheV*, and *thrW* as the most frequently targeted genes (Reiter et al., 1989; Cheetham and Katz, 1995; Williams, 2003). The tDNA pattern targeted by insertions may differ in B2 and A/B1 *E. coli* strains, potentially influencing their ability to acquire and lose PAIs (Escobar-Páramo et al., 2004a; Germon et al., 2007).

Pathogenicity islands often include mobile elements (or fragments thereof), such as bacteriophages, plasmids and insertion sequences. These mobile elements play an important role in recombination, resulting in genetic rearrangements, insertions, deletions and, therefore, variation of PAIs. The elements contribute to the formation of mosaic-like structures, another hallmark of PAIs that promotes the accretion of traits into islands (Dobrindt et al., 2010).

A number of *E. coli* PAIs have the PAI features mentioned above; however, some lack one, two or even more features, making the detection of PAIs from sequenced genomes a challenge. The detection methods usually use the following indicative features of the horizontal origin of GIs and PAIs: (i) biased sequence composition, (ii) gene or motif content (i.e., tRNA/tmRNA, direct repeats, integrases, mobility-related genes, high prevalence of hypothetical proteins and virulence/metabolic/antibiotic resistance genes to subclassify the GIs), and (iii) sporadic phylogenetic distribution assessed by the identification of regions only present in a subset of genomes and/or containing genes usually found in PAIs, such as virulence genes, transposase, integrases or genes coding unknown functions (Langille et al., 2010; Lu and Leong, 2016; Bertelli et al., 2019). Composite detection methods, such as IslandViewer (Bertelli et al., 2017) and GIHunter (Han Wang, 2014), are the most sensitive methods (Bertelli et al., 2019). The data with the highest accuracy are provided by tools such as Islander, which can detect tRNA sequence direct repeats (Hudson et al., 2015). Using these *in silico* approaches, the databases of predicted or curated GIs have been developed from publicly available genome sequences and provide a large sampling of structural variations and gene content in PAIs (Bi et al., 2012; Hudson et al., 2015; Yoon et al., 2015; Bertelli et al., 2017; Li et al., 2018).

## Instability and Motiliy of Pathogenicity Islands

Pathogenicity islands in ExPEC and InPEC can undergo deletion at frequencies ranging from 5 × 10^–3^ to 1 × 10^–6^ (Turner et al., 2001; Tauschek et al., 2002; Middendorf et al., 2004; Bielaszewska et al., 2011). In many cases, excision and integration seem to be mediated by PAI-encoded integrases (Hochhut et al., 2006). These harbor a highly conserved C-terminal domain involved in recombination and a more divergent N-terminal domain that specifically recognizes the integration site *attB* (Figure 1A). During the acquisition, the PAI-specific attachment site *attP* is frequently integrated adjacent to the 3’ end of tDNAs, as observed for phage integration. The recombination results in the direct duplication of the attachment site *attB*, which forms 16 to 130 bp direct repeats (*att*L and *att*R) that flank the PAIs. If multiple isoacceptor tDNAs exist, chromosomal insertion may occur at all available loci, as observed for the high-pathogenicity island (HPI), LEE PAI or PAI II_*J*__96_ (Buchrieser et al., 1998; Tauschek et al., 2002; Bidet et al., 2005). Integrase also excises PAIs from the genome as circular non-replicative intermediates (Figure 1A) using a site-specific recombination process (Rajanna et al., 2003; Hochhut et al., 2006; Wilde et al., 2008). In UPEC strain 536, excision of PAIs I_536_, II_536_ and III_536_ depends on their own integrases. However, PAI V_536_ undergoes excision even in the absence of its integrase, which can be substituted by the integrase of PAI V_536_, suggesting crosstalk between PAIs (Hochhut et al., 2006). Enzymes called excisionases or recombination directionality factors (RDFs) can assist integrases (Lewis and Hatfull, 2001; Sakellaris et al., 2004). A bioinformatic analysis showed that each PAI in UPEC 536 contained its own cognate putative RDF (Napolitano et al., 2011). RDFs act as positive or negative integrase transcriptional regulators and offer stability to their integrase protein partners at the excision site (Numrych et al., 1992; Panis et al., 2010). The mobility of PAI can also be independent of the recombination module and involve homologous DNA recombination (Schubert et al., 2009).

Horizontal transfer (HT) is another aspect of PAI mobility (Figure 1B). Because most islands do not contain an origin of replication and are not able to self-mobilize, HT of excised PAIs has been hypothesized to occur with the help of bacteriophages, ICEs or conjugative plasmids (Middendorf et al., 2004). The presence of phage-related sequences on most PAIs suggests that phages have a key role in HT (Boyd et al., 2001; O’Shea and Boyd, 2002). Alternatively, PAIs can be transferred by conjugation in the presence of an *attB*-presenting helper replicon and accessory transfer genes (Schubert et al., 2009; Schneider et al., 2011). An alternative mechanism, independent of the *att* site, is homologous DNA recombination, which involves sequences shared by plasmids, a PAI or its environment, and the recipient genome (Schubert et al., 2009). The stabilization of beneficial genetic information localized on mobile genetic elements can then be achieved by the selective loss of transfer or mobilization functions encoded by these elements.

## Non-Diarrheagenic *Escherichia coli*

### Physiopathology of ExPEC and AIEC Infections

**FIGURE 2:**
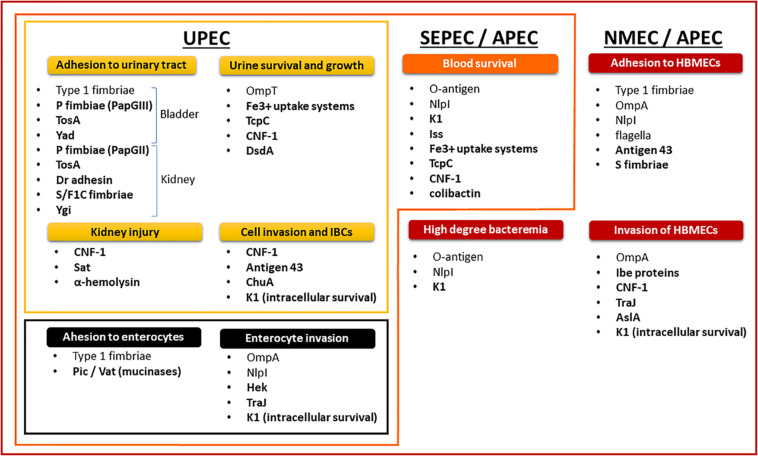
Nesting distribution of pathogenicity factors in ExPEC pathotypes and their role in the physiopathology of infections.

The non-diarrheagenic *E. coli* are opportunistic pathogens. ExPEC take advantage of host behavior and susceptibility by employing virulence factors (Figure 2) to colonize the digestive tract and then move on to the bladder, where they cause cystitis. The cystitis infection can ascend through the ureters to the kidneys, eventually causing pyelonephritis, and potentially reaching the blood compartment to cause sepsis (Johnson, 1991; Nielubowicz and Mobley, 2010). ExPEC use cell-surface adhesins to adhere to the host’s epithelial cells (Croxen and Finlay, 2010). Adhesin-receptor interactions, invasion factors and bacterial engulfment-enhancing toxins stimulate bacterial internalization into apical uroepithelium cells, leading to invading bacteria that are endocytosed into membrane vesicles (Martinez et al., 2000; Eto et al., 2007). The ExPEC then escape from the endocytic vesicles (Mulvey et al., 1998, 2001; Anderson et al., 2003; Eto et al., 2007; Rosen et al., 2007; Flores-Mireles et al., 2015), proliferate inside the cellular cytoplasm, and form intracellular bacterial communities (IBCs) (Mulvey et al., 1998, 2000; Wright et al., 2007; Flores-Mireles et al., 2015).

Intracellular bacterial communities constitute biofilm-like quiescent reservoirs that preserves ExPEC viability and can lead to recurrent UTIs (Eto et al., 2006). Cytosolic *E. coli* also induce cell lysis and exfoliation (Mulvey et al., 1998, 2000). The innate immune response initiated by infected uroepithelium cells and exfoliation clears many bacteria from the urinary tract with the flow of urine, but this also leaves the underlying layers of immature bladder epithelial cells exposed and more susceptible to infection (Mulvey et al., 1998, 2000, 2001; Bower et al., 2005; Wiles et al., 2008). In a small proportion of UTIs, ExPEC will decrease the expression of bladder-targeting adhesins and enhance the expression of both kidney-targeting adhesins and bacterial motility (Flores-Mireles et al., 2015). Adhesion and mobility allow ExPEC to ascend through the ureters to the kidneys, where they cause acute pyelonephritis (Svanborg et al., 2001).

Asymptomatic bacteriuria (ABU) occurs in up to 6% of healthy individuals and in 20% of elderly individuals. ABU-associated *E. coli* persist for months or years without evoking a destructive mucosal host response (Kunin and Halmagyi, 1962; Lindberg, 1975). *E. coli* strains lacking virulence genes and a defect in TLR4 signaling lead to the asymptomatic carrier state (Andersson et al., 1991; Hull et al., 1999; Frendéus et al., 2001; Svanborg et al., 2001; Bergsten et al., 2005; Fischer et al., 2006; Klemm et al., 2006; Ragnarsdóttir et al., 2008; Grönberg-Hernández et al., 2011).

Bacterial destabilization of the renal epithelium and subsequent inflammation result in renal scarring, which acts as a conduit to the bloodstream. One study showed that 91% of *E. coli* strains isolated from the blood and urine of the same hospitalized patients were closely related, suggesting that UPEC is the main origin of septicemias and SEPEC (Vollmerhausen et al., 2014). The strains originating from UTIs can also be found in the guts of the patients (Moreno et al., 2008). Accordingly, overgrowing intestinal ExPEC strains can also translocate through the gut epithelium and survive in mesenteric lymph nodes to enter the bloodstream (Bark et al., 1995; MacFie et al., 1999; Ljungdahl et al., 2000; Owrangi et al., 2018). Throughout their progression, ExPEC use numerous capture systems to find the necessary elements, particularly iron, to aid their growth (Braun, 2003). In the bloodstream, SEPEC protect themselves from the immune system by surface structures and effectors which allow them to escape the bactericidal activity of complement and phagocytosis (Cross et al., 1984; Kim et al., 1992; Kim, 2016). This resistance is a factor in the induction of a high degree of bacteremia (Kim, 2016).

Several *in vivo* studies on NMEC have pointed out a relationship between the magnitude of bacteremia and the development of meningitis (Kim, 2016). *E. coli* binding and the invasion of human brain microvascular endothelial cells (HBMECs) mediated by *E. coli* pathogenicity factors is also a prerequisite (Huang et al., 1995, 1999; Prasadarao et al., 1996; Wang et al., 1999; Hoffman et al., 2000; Zhao et al., 2018). The vacuoles containing NMEC that result from HBMEC invasion evolve as endosomes without fusion with lysosomes, thereby allowing *E. coli* to cross the blood-brain barrier alive (Kim et al., 2003).

Avian pathogenic *E. coli* strains cause avian colibacillosis, a poultry extraintestinal infection. Extensive genetic similarity has been documented between APEC and ExPEC strains (Rodriguez-Siek et al., 2005; Moulin-Schouleur et al., 2006; Johnson et al., 2007, 2012; Mora et al., 2009, 2012). APEC can cause diseases in mammalian infection models, and these diseases mimic human ExPEC infections (Tivendale et al., 2010). Conversely, human sources of ExPEC can cause diseases in avian species. Human ExPEC and APEC therefore share similar pathogenic potential, and some human-associated ExPEC may have evolved from APEC, and vice versa (Skyberg et al., 2006).

Adherent-invasive *E. coli* strains have an increased prevalence in inflammatory bowel disease (IBD). AIEC strains harbor genetic similarity with ExPEC in terms of phylogenetic origin and pathogenicity genotype. However, only 6.3% of ExPEC strains exhibit AIEC phenotypic features, suggesting that the AIEC pathotype is disease specific and contributes to the pathophysiology of chronic IBD (Palmela et al., 2018). AIEC bacteria interact with the intestinal mucosa and can (i) cross the mucus layer and resist antimicrobial peptides, (ii) adhere to the surface of the mucosa and increase intestinal permeability, and (iii) penetrate the lamina propria, persist in macrophage vacuoles and chronically stimulate the immune system. Apart from having a high genotypic variability, AIEC belong mainly to phylogroup B2 and resemble ExPEC in terms of virulence factor content. However, AIEC could have selected an original regulatory system and/or pathoadaptive mutations to enable better adaptation to intestinal conditions.

### PAIs in ExPEC and AIEC

Experimental and epidemiological investigations of ExPEC pathogenicity factors (PFs) show that no PF is limited to, and no single clone is required for, any particular extraintestinal infection. Statistical analyses of PF content and the resulting lethality in an animal model suggested that the virulence of ExPEC is more likely linked to the number of PFs than to the genetic background (Picard et al., 1999; Johnson and Kuskowski, 2000). In addition, the success of pathogens at various host sites can be due to multiple combinations of PFs, and virulence potential probably exists as a continuum that depends on the number and type of PFs and the inoculum size (Russo and Johnson, 2000). The prevalence of PFs in ExPEC infections is reported in Table 1.

**TABLE 1:** Prevalence of virulence factors in ExPEC according to the source (Korhonen et al., 1985; Guyer et al., 2000, 2000; Russo et al., 2001; Bingen-Bidois et al., 2002; Johnson et al., 2002, 2004, 2008; Marrs et al., 2002; Bonacorsi et al., 2003; Russo, 2003; Srinivasan et al., 2003; Buckles et al., 2004; Bonacorsi and Bingen, 2005; Parham et al., 2005a; Rendón et al., 2007; Restieri et al., 2007; Ananias and Yano, 2008; Henderson et al., 2009; Schubert et al., 2010; Ejrnæs et al., 2011; Mahjoub-Messai et al., 2011; Vejborg et al., 2011; Lehti et al., 2012; Qin et al., 2013; Tarchouna et al., 2013; Firoozeh et al., 2014; Basmaci et al., 2015; Nagarjuna et al., 2015; Wijetunge et al., 2015; Beck et al., 2016; Cordeiro et al., 2016; Lee and Lee, 2018; Daga et al., 2019; Nojoomi and Ghasemian, 2019; Raimondi et al., 2019; Sarowska et al., 2019).

DESIGNATION	ADHESIN	FUNCTION AND ASSOCIATED INFECTIONS	Feces (%)	Cystitis (%)	APN (%)	Blood (%)	LCS (%)	PATHOTYPE
Type 1 fimbriae	*fimH*	Colonization factor and biofilm formation in EIs, especially cystitis and meningitidis, by binding receptor D-Mannose	68 to 99	62 to 100	88 to 99	90 to 98	92	UPEC, SEPEC, MNEC, APEC
ECP fimbriae	Mat	Meningitis associated and temperature regulated fimbriae	48	91 to 98	92	100	96	NMEC
Curli fimbriae	*csgAB*	Attachment and invasion of host cells, interaction with host proteins, activation of the immune system, bacterial aggregation and biofilm formation	34 to 36	44 to 100	24	53 to 92	NF	UPEC, SEPEC, APEC
P fimbriae	*papGII*	Proinflammatory activity and colonization factor in EIs especially pyelonephritis by binding GbO4 and GbO3	17 to 23	13	64 to 67	48 to 68	20 to 50	UPEC, SEPEC, MNEC, APEC
	*papGIII*	Proinflammatory activity and colonization factor in EIs especially cystitis by binding receptors GbO5 and GloboA	8 to 9	22	19 to 35	14 to 27	1 to 6	UPEC, SEPEC, APEC
S fimbriae	*sfaS*	Adhesion to intestinal and urinary tract cells and facilitate penetration into the tissues by binding receptors NeuNAc (α2-3)Gal in UTIs, meningitidis	7 to 32	13 to 34	88 to 99	39 to 97	28 to 59	UPEC, SEPEC, NMEC
F1C fimbriae	*focG*	Adhesion to epithelial and endothelial cells in the bladder and kidney	3 to 6	14 to 39	35	21	1 to 4	UPEC, SEPEC
G fimbriae	*gafD*	Binding GlcNAc receptor	5	0 to 4	0 to 18	0	3	
Auf fimbriae	*auf*	Auf fimbriae	21 to 27	67	NF	NF	62	UPEC, NMEC
RTX protein TosA	*tosA*	Adhesion to host cells derived from the upper urinary tract, enhances survival in disseminated infections and lethality during sepsis	11	16	29			UPEC
Dr fimbriae	*dra*	Binding to the receptors DAF of epithelial cells, type IV collagen and CEACAMs. Promotes host cells invasion	6	0 to 4	6 to 12	5	NF	UPEC
Afimbrial adhesin	*afa*	Binding to receptor DAF on the epithelial cells and hemagglutination capacity	2 to 17	19 to 81	16	49	9	UPEC
IrgA homolog adhesin	*iha*	Iron-regulated-gene-homolog adhesin	14 to 24	22	41	33 to 40	19	UPEC, SEPEC
DESIGNATION	INVASIN	FUCNTION AND ASSOCIATED INFECTIONS	Feces (%)	Cystitis (%)	APN (%)	Blood (%)	LCS (%)	PATHOTYPE
hemagglutinin	*hek/hra*	Promote autoaggregation, hemagglutination, epithelial cell invasion and is associated with bacteremia in newborns	14 to 28	28	33 to 66	45	11	UPEC, SEPEC, NMEC, APEC
Endothelial brain invasion	*ibeA*	Cell invasion into the host tissues such as endothelial cell by binding Vimentine and Caspr1 to promote penetration of the blood–brain barrier	1 to 15	13 to 23	29	4 to 11	32 to 38	UPEC, NMEC, SEPEC, APEC
Arylsulfatase	*aslA*	Contributes to brain microvascular endothelial cell invasion	46	NF	NF	NF	87 to 89	NMEC
DESIGNATION	TOXINS	FUCNTION AND ASSOCIATED INFECTIONS	Feces (%)	Cystitis (%)	APN (%)	Blood (%)	LCS (%)	PATHOTYPE
α-hemolysin	hly	Pore-forming RTX toxin responsible for kidney injury and inflammation	4 to 15	15 to 43	38 to 47	34 to 77	9 to 30	UPEC, NMEC
Cytotoxic necrotizing factor 1	cnf1	activation of Rho GTPases, engaging in cell necrosis and promoting resistance to phagocytosis, inflammation, recurrent UTIs and blood-brain barrier penetration	3 to 13	13 to 39	28 to 47	17 to 49	6 to 27	UPEC, SEPEC, NMEC
Cytolethal distending toxin	cdt	DNAse inducing apoptosis and cellular senescence resulting in cell distention	0 to 1	9 to 17	12	0 to10	46	SEPEC
Colibactin	clb	PK-NRP compounds inducing DNA damage resulting preferentially in apoptosis in immune cells and premature cellular senescence in epithelial cells.	11 to 32	33	44	18 to 58	75	UPEC, SEPEC, NMEC, APEC
DESIGNATION	ATs	FUCNTION AND ASSOCIATED INFECTIONS	Feces (%)	Cystitis (%)	APN (%)	Blood (%)	LCS (%)	PATHOTYPE
Antigen43	*agn43*	Autotransporter involved in auto-aggregation, adhesion to host cells, biofilm development and persistence	41	29 to 94	65	69	NF	UPEC
Sap	sap	Autotransporter adhesin	NF	1,5	NF	NF	NF	
Secreted autotransporter toxin	Sat	Autotransporter having serine protease activity inducing cytotoxic effect and kidney injury	14 to 24	23 to 56	55 to 68	26 to 39	49	UPEC, NMEC
Vacuolating autotransporter toxin	Vat	Autotransporter having serine protease activity affecting mucins, facilitating epithelium colonization and inducing host cell vacuolization	0 to 49	42 to 68	44	12 to 71	51 to 76	UPEC, APEC, NMEC
Temperature-sensitive hemagglutinin	tsh	Autotransporter having temperature-sensitive hemagglutinin activity	1 to 5	4 to 8	18	20	11	APEC, NMEC
Serin protease autotransporter	pic	Autotransporter exhibiting serine protease activity affecting mucins, facilitating epithelium colonization and damaging host cell membrane	0 to 23	21 to 34	14 to 40	0 to 36	NF	UPEC
Secreted and surface associated lipoprotein	sslE/yghJ	Cytotoxic metalloprotease exihiting mucinase and proinflammatory activity	NF	NF	NF	NF	NF	NMEC,IPEC
Contact-dependent growth inibition	cdiAB	Induce the growth inhibition of the bacterial target	NF	NF	NF	NF	NF	UPEC

**DESIGNATION**	**IRON UPTAKE**	**FUNCTION AND ASSOCIATED INFECTIONS**	**Feces (%)**	**Cystitis (%)**	**APN (%)**	**Blood (%)**	**LCS (%)**	**PATHOTYPE**

Enterobactin	ent, fepA	Acquisition of siderophore Enterobactin-Fe3 +	100	100	*	*	*	UPEC, SEPEC, MNEC, APEC
Aerobactin	aer, iuc, iut	Acquisition of siderophore Aerobactin-Fe ions in the host involved in UTI	16 to 41	24 ot 52	38 to 77	54 to 80	61 to 88	UPEC, APEC, NMEC
Salmochelin	iroN	Acquisition of siderophore Salmochelin-Fe ions in the host involved in UTI and meningitidis	5 to 26	33 to 74	76 to 78	44 to 63	38 to 75	UPEC, NMEC, SEPEC APEC
Yersiniabactin	irp, fuyA	Acquisition of siderophore Yersiniabactin-Fe ions in the host involved in UTI and meningitidis	23 to 70	63 to 96	97	82	90 to 99	NMEC
ChuA, Hma	chu, hma	Acquisition of Fe from hemoglobin in the host system	53 to 59	66 to 80	66 to 71	48 to 73	89	UPEC, SEPEC
Sit	sitDCBA	Transportation of Fe and Mn	25	44	56	96	92	UPEC, APEC
IreA	ireA	Iron-regulated homolog of siderophore receptors involved in UTI	7 to 19	13	47	32	NF	UPEC
Fec	fecARI	Acquisition of citrate-Fe3 +	NF	NF	NF	NF	NF	
Fbp	fbpABCD	Fe acquisition	NF	58	59	45	NF	
K1 capsule	*kpsMI-neuA*	Enable resistance to complement activation and phagocytosis, immunological tolerance and intracellular survival promoting both bacteremia and meningitis	0 to 27	35	13 to 47	8 to 82	75 to 90	SEPEC, NMEC
TcpC	tcpC	Impairs the innate immune response by inhibiting Toll-like receptor and MyD88-specific signaling	8	22	42	32 to 39	0	UPEC, SEPEC
D-serine deaminase	dsdCXA	Promotes bacterial growth by using D-serine as carbon, nitrogen, and energy source, and prevent the bacteriostatic activity of D-serine in the urine and brain.	0 to 4	20 to 22	NF	6	73 to 87	UPEC, NMEC
TrapT	traT	Inhibition of the classical pathway of complement activity and serum survival	43 to 88	36 to 68	49 to 81	50 to 81	NF	UPEC, SEPEC
	Iss	Resistance to complement and serum survival	4 to 20	13	35	10 to 34	25	UPEC, SEPEC, NMEC
Outer membrane protein T	OmpT	Resistance to protamine and urine survival	15 to 68	70	88 to 95	21 to 81	96	UPEC, SEPEC, NMEC

*NF, data not found, ^∗^Present in almost all *Escherichia coli* genomes.*

**FIGURE 3:**
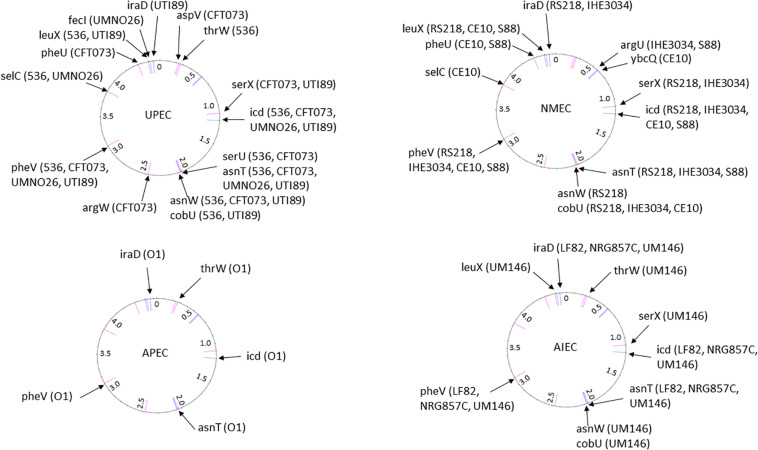
The position of PAI insertion sites in the non-diarrheagenic *E. coli* pathotypes. The name of genetic features associated with the insertion is indicated with the name of bacterial strains harboring PAI at the corresponding site. The position of the insertion site is indicated in pink for tRNA genes and in blue for coding genes. The numbers reported in the inner ring are the position (megabase) in the genome of the non-pathogenic *E. coli* strain MG1565 used as a reference.

Most ExPEC PFs are encoded by PAIs (Table 2), and the encoded functions are involved in critical steps of the infectious process. The cooperative effect of PAIs has been experimentally confirmed with the prototype strains 536, CFT073 and RS218 (Silver et al., 1980; Hacker et al., 1983; Brzuszkiewicz et al., 2006) by monitoring UTI, septicemia and meningitis mouse models using single-PAI and/or multiple-PAI deletion mutants (Schubert et al., 2002; Xie et al., 2006; Lloyd et al., 2009a; Tourret et al., 2010). These studies showed an additive contribution of PAIs to extraintestinal virulence, but redundancies (i.e., iron uptake) were also evident. The involvement of PAIs in virulence also depends on the genetic background and on the infection model used to test them, suggesting a complex network of pathogenetic possibilities resulting from multiple and independent acquisitions of functions without an unequivocal goal. Given the functional redundancies in PAIs, and to provide an overview of the pathogenicity functions encoded by ExPEC PAIs, their PF content is presented here by PF type and the main structural features of corresponding PAIs is reported in Table 2 and Figure 3 for reference strains.

**TABLE 2:** Islands predicted by IslandViewer and/or genomic comparison to *E. coli* K12 from sequenced strains belonging to pathotypes UPEC (536, CFT073, UMNO26, and UTI89), NMEC (RS218, IHE3034, CE10, and S88), APEC (APECO1) and AIEC (LF82, NRG857C, and UM146) and commensal strains (ED1a and HS).

PAI name (alternatives)	Chromosomal insertion site	Size (kb)	%GC	Virulence/fitness factors within islands (≥4kb)	Position
**UPEC strain 536 (Genbank accession number: NC_008253, 4938920-bp)**					
PAI-536-thrW (III_536_)	*thrW*	77	47	S pilus, Salmochelin, Ag43, Vat	294409.371141
PAI-536-icd	*icd*	47	50	Iss, Sit system	1187870.1235252
PAI-536-serU (VII_536_)	*serU*	22	38	TcpC	1947667.1969721
PAI-536-asnT (IV_536_, HPI)	*asnT*	30	57	Yersiniabactin	1971318.2001603
PAI-536-asnW (VI_536_)	*asnW*	54	53	Colibactin	2015441.2068949
PAI-536-cobU	*cobU*	43	49	Hma, Fbp system	2075300.2116007
PAI-536-pheV (V_536_)	*pheV*	80	48	Pix pilus, Ag43, K15 capsule	3128357.3208357
PAI-536-selC (I_536_)	*selC*	77	46	α-Hemolysin, PapX, F17-like pilus, CS12-like pilus	3948173.4025637
PAI-536-leuX (II_536_)	*leuX*	102	46	Hek, P pilus, α-Hemolysin, CdiAB, DsdA	4735418.4840138
**UPEC strain CFT073 (Genbank accession number: NC_004431, 5231428-bp)**					
PAI-CFT073-aspV (III_*CFT*__073_)	*aspV*	100	47	Pic, Hma, TosA, fbp system	248751.348573
PAI-CFT073-serX	*serX*	113	49	S pilus, Salmochelin, Ag43	1127702-1241149
PAI-CFT073-icd	*icd*	54	50	Sit system	1397370.1450849
PAI-CFT073-serU	*serU*	22	38	TcpC	2194512.2216563
PAI-CFT073-asnT (HPI)	*asnT*	32	57	Yersiniabactin	2218159.2250547
PAI-CFT073-asnW	*asnW*	54	53	Colibactin	2262986.2316960
PAI-CFT073-cobU	*cobU*	43	49	Hma, Fbp system	2322324.2365760
PAI-CFT073-argW	*argW*	17	44	IpuA, IpuB, DsdA	2747237.2764564
PAI-CFT073-pheV (I_*CFT*__073_)	*pheV*	123	47	α-Hemolysin, P pilus, Iha, Sat, Aerobactin, Ag43, K2 capsule	3406498.3529292
PAI-CFT073-pheU (II_*CFT*__073_)	*pheU*	52	48	P pilus, IreA	4919569.4971387
**UPEC strain UMNO26 (Genbank accession number: NC_011751, 5202090-bp)**					
PAI-UMNO26-icd	*icd*	46	50	Iss, Sit system	1411940.1457673
PAI-UMNO26-asnT (HPI)	*asnT*	66	52	Yersiniabactin, T4SS	2277413.2343149
PAI-UMNO26-pheV	*pheV*	116	47	P pilus, Iha, Sat, Aerobactin, Ag43, K5 capsule	3445979.3561885
PAI-UMNO26-selC	*selC*	17	48	eaeX	4301079.4320994
PAI-UMNO26-fecI	*fecI*	26	50	Ag43	5031002.5057400
**UPEC strain UTI89 (Genbank accession number: NC_007946, 5065741-bp)**					
PAI-UTI89-serX	*serX*	62	48	S pilus, Salmochelin, Ag43	1080842.1142536
PAI-UTI89-icd	*icd*	47	50	Iss, Sit system	1242292.1288830
PAI-UTI89-asnT	*asnT*	31	57	Yersiniabactin	2069935.2101331
PAI-UTI89-asnW	*asnW*	54	53	Colibactin	2113982.2168375
PAI-UTI89-cobU	*cobU*	42	49	Hma, Fbp system	2173987.2216153
PAI-UTI89-pheV	*pheV*	18	40	K1 capsule	3288044. 3306044
PAI-UTI89-leuX	*leuX*	122	46	Hek, P pilus, F17-like pilus, CNF1, α-Hemolysin, CdiB, DsdA	4779711.4900870
PAI-UTI89-iraD	*iraD*	20	46	IbeA	4919657.4940004
**NMEC strain RS218 (Genbank accession number: CP007149, 5087638-bp)**					
PAI-RS218-serX	*serX*	62	48	S pilus, Salmochelin, Ag43	1060280.1123164
PAI-RS218-icd	*icd*	49	50	Iss, Sit system	1219583.1268928
PAI-RS218-asnT (HPI)	*asnT*	31	57	Yersiniabactin	2089060.2120469
PAI-RS218-asnW	*asnW*	54	53	Colibactin	2133298.2134569
PAI-RS218-cobU	*cobU*	43	49	Hma, Fbp system	2191418.2235286
PAI-RS218-pheV	*pheV*	18	40	K1 capsule	3307311.3325311
PAI-RS218-leuX	*leuX*	123	46	Hek, P pilus, F17-like pilus, CNF1, α-Hemolysin, CdiB, DsdA	4841086.4963833
PAI-RS218-iraD (GimA)	*iraD*	21	46	IbeA	4982442.5003432
**NMEC strain IHE3034 (Genbank accession number: NC_017628, 5108383-bp)**					
PAI-IHE3034-argU	*argU*	50	50	Iss, ompT	559677.609271
PAI-IHE3034-serX	*serX*	62	48	S pilus, Salmochelin, Ag43	1123943.1185632
PAI-IHE3034-icd	*icd*	48	50	Iss, Sit system	1285251.1331958
PAI-IHE3034-asnT (HPI)	*asnT*	31	57	Yersiniabactin	2148149.2179443
PAI-IHE3034-cobU	*cobU*	43	49	Hma, Fbp system	2252158.2294325
PAI-IHE3034-pheV	*pheV*	18	46	K1 capsule	3441108.3459812
PAI-IHE3034-iraD (GimA)	*iraD*	21	46	IbeA	4964058 4984404
**NMEC strain CE10 (Genbank accession number: NC_017646, 5313531-bp)**					
PAI-CE10-ybcQ	*ybcQ*	32	52	Iss	574012.606099
PAI-CE10-icd	*icd*	56	52	Sit system	1285000.1341381
PAI-CE10-asnT (HPI)	*asnT*	31	57	Yersiniabactin	2259714.2291113
PAI-CE10-cobU	*cobU*	27	47	Hma	2309373.2336831
PAI-CE10-pheV	*pheV*	63	46	P pilus, Aerobactin, Sat, Iha, K1 capsule	3487305.3550576
PAI-CE10-selC	*argW*	10	42	IpuA, IpuB (DsdA)	4303582.4357800
PAI-CE10-pheU	*pheU*	20	47	Hek, P pilus	4961804.4982152
PAI-CE10-leuX	*leuX*	42	49	Fec, VirK, MsbB2	5127122.5169094
**NMEC strain S88 (Genbank accession number: NC_011742, 5032268 bp-bp)**					
PAI-S88-argU	*argU*	38	52	ompT	574349.612629
PAI-S88-icd	*icd*	48	50	Iss, Sit system	1183142.1229738
PAI-S88-asnT (HPI)	*asnT*	31	57	Yersiniabactin	1998347.2029756
PAI-S88-pheV	*pheV*	74	45	Hek, P pilus, IreA, K1 capsule	3219875.3293447
PAI-S88-pheU	*pheU*	64	48	PapX	4621378.4684940
PAI-S88-leuX	*leuX*	39	50	Fec, Ag43	4821000.4862061
**APEC strain APECO1 (Genbank accession number: NC_008563, 5082025-bp)**					
PAI-APECO1-thrW	*thrW*	20	41	Tsh	295124.315041
PAI-APCEO1-icd	*icd*	49	50	Iss, Sit system	1179544.1226126
PAI-APECO1-asnT	*asnT*	31	57	Yersiniabactin	2090794.2122085
PAI-APECO1-pheV	*pheV*	73	44	Hek, P pilus, IreA, K1 capsule	3305091.3378655
PAI-APECO1-iraD (GimA)	*iraD*	21	46	IbeA	4935886.4956233
**AIEC strain LF82 (Genbank accession number: NC_011993, 4773108-bp)**					
PAI_LF82-icd	*icd*	49	50	Iss, Sit system	1174302 1223227
PAI-LF82-asnT	*asnT*	31	57	Yersiniabactin	2007750 2039203
PAI-LF82-pheV	*pheV*	18	42	K5 capsule	3110301 3128705
PAI-LF82-iraD (GimA)	*iraD*	21	46	IbeA	4673091 4693405
**AIEC strain NRG857C (Genbank accession number: NC_017634, 4747819-bp)**					
PAI-NRG857C-icd	*Icd*	61	50	Iss, Sit system	1171022 1231985
PAI-NRG857C-asnT	*asnT*	31	57	Yersiniabactin	2014814 2046266
PAI-NRG857C-pheV	*pheV*	18	42	K5 capsule	3084523 3102935
PAI-NRG857C-iraD (GimA)	*iraD*	21	46	IbeA	4648143 4668457
**AIEC strain UM146 (Genbank accession number: NC_017632, 4993013-bp)**					
PAI-UM146-pheV	*pheV*	28	46	K1 capsule	326687 354390
PAI-UM146-cobU	*cobU*	42	49	Hma, Fbp system	1426242 1468411
PAI-UM146-asnW	*asnW*	54	53	Colibactin	1474024 1529464
PAI-UM146-asnT	*asnT*	31	57	Yersiniabactin	1541958 1573422
PAI-UM146-icd	*icd*	29	53	Sit system	2353562 2382356
PAI-UM146-serX	*serX*	62	48	S pilus, Salmochelin, Ag43	2499854 2561497
PAI-UM146-thrW	*thrW*	20	41	Tsh	3301638 3321999
PAI-UM146-leuX	*leuX*	121	46	Hek, P pilus, F17-like pilus, CNF-1, α-Hemolysin, CdiA, DsdA	4520979 4642133
PAI-UM146-iraD (GimA)	*iraD*	20	46	IbeA	4660918 4681265
**Commensal strain ED1a (Genbank accession number: NC_011745, 5209548-bp)**					
PAI-ED1a-icd	*icd*	12	49	Sit system	1282697.1294467
PAI-ED1a-asnT (HPI)	*asnT*	31	57	Yersiniabactin	2164139.2222325
PAI-ED1a-pheU	*pheU*	44	48	Iha, Aerobactin	4837580 4881090
PAI-ED1a-leuX	*leuX*	57	50	MsbB2, VirK, fec, Ag43	5065910.5122732
**Commensal strain HS (Genbank accession number: NC_009800, 4643538-bp)**					
PAI-HS-hisF	*hisI*	39	47	Klebsiella-related LPS and capsule determinants	2143083.2181967

#### Adhesins Encoded or Regulated by PAIs

The capacity of *E. coli* to adhere to host cells plays a fundamental role in the colonization of any site. Adhesion is mediated by proteins called adhesins, which specifically recognize receptors and are often contain antigenic sugars that are present on the surface of host cells. Adhesins are exposed directly to the surface of bacteria or are carried by filamentous structures called “fimbriae” or “pili” (Table 1). Most adhesins are encoded by PAIs, except for conserved fimbriae, such as type 1 fimbriae, curly fibers, outer membrane protein A (OmpA), chitin-binding domain of chiA and new lipoprotein I (NlpI) that are involved in the adhesion of AIEC and NMEC to enterocytes and HBMECs (Kim, 2003, 2008).

Type 1 fimbriae are present in more than 80% of *E. coli* strains and other Enterobacteriales (Abraham et al., 1988). However, they are major players in ExPEC virulence, and their expression is regulated by PAIs. The type 1 fimbria adhesin FimH binds specifically to α-D-mannose residues attached to membrane glycoproteins exposed at the surface of bladder cells, enterocytes and the endothelial cells of brain capillaries (Keith et al., 1986; Teng et al., 2005). The role of type 1 pili in cystitis has been demonstrated by mutagenesis and in animal models (Keith et al., 1986; Johnson, 1991; Connell et al., 1996). Their adhesin FimH binds to glycosylated uroplakin and α1β3 integrins covering the apical surface of uroepithelium cells, thereby promoting the invasion of host cells and the development of IBCs (Connell et al., 1996; Martinez et al., 2000; Gunther et al., 2002; Mysorekar and Hultgren, 2006; Eto et al., 2007; Flores-Mireles et al., 2015). Notably, in some AIEC, UPEC, MNEC and APEC strains, the protein sequence of FimH adhesin has polymorphisms that confer a greater ability to interact with D-mannose.

The expression of type 1 pili depends on a promoter encoded by the phase-variation invertible element *fimS*. The orientation of *fimS* can be reversed by the recombinases FimB and FimE encoded within the operon *Fim*, and by recombinases FimX, IpuA and IpbA encoded by PAIs (Klemm, 1986; McClain et al., 1991; Bryan et al., 2006; Hannan et al., 2008). IpbA is ubiquitous, as it is found approximately half the time in both commensal fecal and uropathogenic isolates of *E. coli* (Bryan et al., 2006). FimX-encoded PAI is observed in more than 80% of UPEC and is more prevalent in the UPEC of a lower urinary tract origin (87.5%) than of upper urinary tract origin (74%) or of commensal isolates (36%) (Bryan et al., 2006; Bateman et al., 2013). IpuA-encoding PAIs also tend to occur more frequently among UPEC strains than in commensal fecal isolates (Bateman et al., 2013). This observation is consistent with the hypothesis that FimW and IpuA recombinases play a role in the regulation of *E. coli* uropathogenesis. FimX, IpuA and IpbA promote inversion of *fimS* from OFF to ON and probably promote bladder colonization, while FimE inverts the promoter from ON to OFF and could therefore promote the release of bacteria from the bladder epithelium and the infection of the higher tract. The latter might be enhanced by a coordinated upregulation of both bacterial mobility and other adhesins, such as P fimbriae (Klemm, 1986; McClain et al., 1991; Snyder et al., 2005; Bryan et al., 2006; Hannan et al., 2008).

FimX also regulates the predicted LuxR-like response regulator HyxR, which is encoded from the same PAI and is a negative regulator of the nitrosative stress response and intracellular macrophage survival (Bateman and Seed, 2012b,a). IpuA and the associated recombinase IpuB, which lacks activity on *fimS*, regulate the orientation of a phase-variable invertible element *ipuS*, which is located in the same PAI, proximal to *ipuA* and *ipuB* (Battaglioli et al., 2018). The orientation of *ipuS* drives the transcription of a two-gene operon containing *ipuR* (a predicted LuxR-type regulator) and *upaE* (an autotransporter involved in biofilm formation and extracellular matrix adhesion) (Battaglioli et al., 2018). Consistent with this phenotype, the *ipuS* orientation ON results in (i) defective swimming motility, (ii) an increase in adhesion to human kidney epithelial cells, and (iii) promotion of kidney colonization in experimental UTI mouse models (Battaglioli et al., 2018). Overall, therefore, PAIs not only encode PFs, but they are also involved in virulence regulation networks.

P fimbriae are encoded by the *pap* operon (pyelonephritis associated pili), which is associated with different PAIs (Lane and Mobley, 2007). The non-structural gene *papX* of the *pap* operon is another example of the PAI gene that is involved in virulence regulation. The protein PapX binds directly to the *flhDC* promoter, thereby inhibiting the transcription of the master regulator of flagellar biosynthesis, motility and chemotaxis (Li et al., 2001; Simms and Mobley, 2008; Reiss and Mobley, 2011; Reiss et al., 2012). The fimbrial-tip adhesin of the P fimbria PapG (Källenius et al., 1980) binds to glycosphingolipids containing the digalactoside Gal(α1-4β) Gal moieties found in the renal epithelium, P blood-group antigen and gut epithelium (Antão et al., 2009). Three major alleles of the *papG* locus (GI to III) have been described, with alleles GII and GIII being the most frequently encountered in human disease (Marklund et al., 1992). Allele GII recognizes globotriosylceramide (GbO3) and globotetraosylceramide (GbO4),which are particularly abundant in the kidneys, and it is preferentially found in strains isolated from pyelonephritis because allele GIII recognizes globopentaosylceramide (GbO5) and is mainly found in strains isolated from cystitis (Karr et al., 1989; Lindstedt et al., 1991; Marrs et al., 2002). GbO5 is found in the urothelium of animals but is not present in humans, except in certain individuals expressing an analog (GloboA) and who are particularly susceptible to lower UTIs with PapGIII strains (Lindstedt et al., 1991).

PAI-CFT073-*aspV* harbors the *tos* operon, which encodes the repeat-in-toxin (RTX) family member TosA (Lloyd et al., 2009a). The RTX protein family members can be involved in a range of functions, including pore and biofilm formation and adhesion to host cells (Linhartová et al., 2010; Dhakal and Mulvey, 2012). The presence of *tosA* promotes bladder and kidney colonization in a UTI mouse model and is a marker of UPEC (Vigil et al., 2011b,a). Localized to the bacterial surface, TosA mediates adhesion to host cells derived from the upper urinary tract, increases bacterial survival in disseminated infections and enhances lethality during sepsis (Vigil et al., 2012). The TosCBD proteins mediate the production and export of TosA, while TosE and TosF suppress motility by an unknown regulatory function. TosR is a member of the PapB family of transcriptional regulators, including the fimbria-associated regulators PapB and FocB (Xia et al., 1998; Lindberg et al., 2008; Hultdin et al., 2010). TosR regulates the *tos* operon (Engstrom and Mobley, 2016) and other genes involved in adhesion (P, F1C, and Auf fimbriae) and biofilm formation (Luterbach et al., 2018).

Other fimbrial gene clusters are found in ExPEC PAIs (Table 2). F1C/S fimbriae promote adhesion to primary human renal proximal-tubular cells by binding to α-sialyl-2-3-β-galactoside (Kreft et al., 1995). The F1C/S fimbriae are frequently found in NMEC and could also be involved in the crossing of the blood-brain barrier, as occurs with type 1 fimbriae (Korhonen et al., 1985; Ott et al., 1986). However, in a meningitis animal model, the deletion of the F1C/S fimbriae operon in *E. coli* K1 did not significantly affect bacterial binding to and invasion of HBMECs, and the deletion also did not affect bacterial penetration into the central nervous system (Kim, 2008). Ygi fimbriae promote renal tropism, biofilm formation and *in vivo* fitness in the urine and kidneys (Spurbeck et al., 2011). Yad fimbriae are necessary for adhesion to a bladder epithelial cell line and biofilm formation, and the deletion of these fimbrial genes has been shown to increase motility (Spurbeck et al., 2011). A double deletion strain, Δ*ygi* Δ*yad*, showed impaired colonization of the urine, bladder, and kidneys in a mouse model, demonstrating that these fimbriae contribute to uropathogenesis (Spurbeck et al., 2011). ExPEC also produces non-fimbrial adhesin FdeC that is highly prevalent in both commensal strains and InPEC. This adhesin binds epithelial cells and, collagen V and VI, and *in vivo* experiments suggest that it has a role in UTI infections (Nesta et al., 2012).

Overall, PAIs provide the ability to produce multiple fimbria that confer a selective advantage within particular niches and are involved in crosstalk between regulators of various fimbrial types and in motility.

#### Invasins Encoded by PAIs

Extraintestinal *E. coli* PAIs can produce invasins, which are structures that promote bacterial internalization within host cells. This process allows bacteria to escape targeting by the immune system and is involved in the formation of the IBCs observed in UTIs and Crohn’s disease with UPEC and AIEC, respectively. These internalizations also allow bacteria to cross epithelial and endothelial barriers by transcytosis.

The PAI-encoded protein Hek/Tia, which is more frequently found in UPEC than in commensal isolates (Table 1), is responsible for bacterial aggregation on the surface of enterocytes, as is observed for enteroaggregative *E. coli*, and is involved in the invasion of T84 enterocytic cells (Fagan and Smith, 2007). The mechanism behind the invasion of enterocytes and the bladder epithelium and the requirement for preliminary adhesion are still largely unknown. The invasion of HBMECs by NMEC requires binding to and the invasion of the HBMECs and involves the PAI-encoded microbial factors IbeA and cytotoxic necrotizing factor-1 (CNF-1), as well as the housekeeping factors OmpA, NlpI and arylsulfatase-like gene *aslA* (Huang et al., 1995, 1999, 2001; Prasadarao et al., 1996; Wang et al., 1999; Hoffman et al., 2000; Khan et al., 2002; Kim, 2003, 2008). Studies have attempted to identify the receptors at the surface of HBMECs for these bacterial factors: CNF-1 interacts with laminin receptors, while IbeA binds to both vimentin and HBMEC contactin-associated protein 1 (CASPR1) (Zou et al., 2006; Zhao et al., 2018). This latter interaction activates focal adhesion and kinase signaling, which causes *E. coli* internalization (Zhao et al., 2018).

#### Toxins Encoded by PAIs

Extraintestinal *E. coli* strains can use PAI-encoded genes to produce several toxins, including α-hemolysin, CNF-1, cytolethal and distending toxin (Cdt), and colibactin (Clb).

*E. coli* α-hemolysin is a pore-forming toxin belonging to the class of RTX toxins that are encoded by the *hly* operon. Although most UPEC strains carry one copy of the hemolysin operon, pyelonephritogenic strains 536 and J96 harbor two copies of the *hly* operon and both loci are required for full virulence (Nagy et al., 2006). The toxin is encoded by the *hlyA* gene. The gene *hlyC* encodes an acyl transferase that is required for activation of the toxin, while genes *hlyB* and *hlyD* are involved in the energy-dependent secretion of HlyA (Welch, 1991). HlyA is a hemolysin that targets different cell types, including leukocytes (Galmiche and Boquet, 2001). The involvement of α-hemolysin in virulence has been suggested because of its high prevalence in UPEC compared to its prevalence in fecal strains and because of its high expression within IBCs (Johnson, 1991; Reigstad et al., 2007). In upper urinary tract infections, α-hemolysin is thought to play both a direct cytotoxic role in renal cells and a pro-inflammatory role that causes the secretion of cytokines IL-6 and IL-8 and alters Ca^2+^ membrane flow (Uhlén et al., 2000). The combination of these two actions weakens the renal epithelium and promotes the passage of bacteria into the blood (Bonacorsi et al., 2006). By interfering with NF-kB-mediated proinflammatory signaling pathways and triggering the breakdown of paxillin and other host regulatory proteins, HlyA also dampens the host’s immune response to infection and enhances the exfoliation of bladder epithelial cells (Dhakal and Mulvey, 2012).

CNF-1 induces the formation of giant multinucleated cells, changes actin and tubulin organization, and most likely promotes cell spreading (Fiorentini et al., 1988). Its toxic activity arises because of the post-translational activating mutation of Rho GTPases by deamidation, an essential control factor in the shape, adhesion, mobility, phagocytosis, and oxidative burst in host cells (Flatau et al., 1997). The CNF-1 prevalence is higher in strains isolated from UTIs and prostatitis than in fecal isolates (Caprioli et al., 1987; Bingen-Bidois et al., 2002). An animal model of UTIs has shown an involvement of CNF-1 in resistance to phagocytosis by macrophages and in the induction of deep and persistent infections of the bladder (Rippere-Lampe et al., 2001). The CNF-1-mediated disruption of the Rho GTPase signaling pathways has antiapoptotic abilities in uroepithelium cells and leads to immune dysregulation, while conferring a survival advantage in the presence of neutrophils (Davis et al., 2005). CNF-1 also plays an important role in the rearrangement of the HBMEC cytoskeleton that allows crossing of the blood-brain barrier (Badger et al., 2000; Kim, 2002).

The *pks* genomic island present in *E. coli* strains of phylogroup B2 encodes colibactin, a hybrid polyketide/non-ribosomal peptide that causes DNA damage and cell cycle arrest of eukaryotes (Nougayrède et al., 2006). The colibactin-encoding determinant has been detected primarily in extraintestinal pathogenic isolates of *E. coli*, other Enterobacteriales, and commensal *E. coli* (Nougayrède et al., 2006; Putze et al., 2009). *E. coli* persisting in the infant gut microbiota tends more often to carry the *pks* island than do either intermediate-term colonizers or transient strains, suggesting that the *pks* island contributes to the gut-colonizing capacity of group B2 strains (Nowrouzian and Oswald, 2012). The frequent detection of the *pks* island in *E. coli* isolated from biopsies of patients suffering from colon cancer also suggests involvement in long-term intestinal colonization, raising the question of its role in colorectal cancer (Swidsinski et al., 1998; Martin et al., 2004; Bronowski et al., 2008; Cuevas-Ramos et al., 2010; Arthur et al., 2012; Buc et al., 2013; Cougnoux et al., 2014, 2016; Pleguezuelos-Manzano et al., 2020). The presence of the *pks* island in ExPEC could also indicate that colibactin contributes to fitness or virulence during extraintestinal infections (Johnson et al., 2008; Krieger et al., 2011; McCarthy et al., 2015). A recent observation indicates that the probiotic effects of the *E. coli* Nissle 1917 strain to ameliorate colitis severity and to modulate cytokine expression cannot be separated from the strain’s ability to express functional colibactin (Olier et al., 2012). This finding demonstrates that, depending on the niche or context, colibactin-producing PAI can be considered either a virulence factor and/or a probiotic factor.

The TLRs are an important family of innate sensors that recognize diverse microbial products and launch the signaling pathways that ultimately lead to the clearance of the pathogen from the host and the establishment of a memory response in anticipation of any subsequent attack. ExPEC PAI-*serU* can interfere directly with the TLR signaling pathway by producing an inhibitor homolog of TLR receptors to dampen the NF-kB-induced proinflammatory response (Cirl et al., 2008). This protein, called the TLR domain containing-protein C (TcpC), is secreted by an efflux pump and then internalized into macrophages, where it can disable TLR signaling through direct binding of MyD88, the key TLR signaling adaptor (Cirl et al., 2008). The *tcpC* gene is present in 30–40% of the *E. coli* strains isolated from septicemia and in almost half of those causing pyelonephritis but is less common in cystitis (22%), ABU (16%) and *E. coli* strains of the fecal microbiota (8%) (Table 1). TcpC can increase the severity of UTIs in humans, which is consistent with the important role of TLR4 in host defense in the urinary tract (Samuelsson et al., 2004).

#### Autotransporters Encoded by PAIs

Extraintestinal *E. coli* PAIs encode several autotransporters (ATs), which are a family of proteins that mediate their own secretion through the outer membrane of gram-negative bacteria. Also known as the type V secretion system, ATs contain a C-terminal membrane anchor region that forms a pore through which the passenger domain is translocated to the cell surface. The passenger domain of ATs is exposed at the bacterial surface and/or secreted after autoproteolytic cleavage. The AT passenger domains are diverse in function and can act as enzymes (lipases, esterases, or proteases) and/or as adhesins (Tapader et al., 2019).

AT antigen 43 (Ag43) is widely distributed among *E. coli* strains, including UPEC. Ag43 mediates autoaggregation, biofilm formation and host cell adhesion. It promotes persistent colonization in a UTI mouse model and is expressed within IBCs, suggesting that Ag43 plays a role in the intracellular growth phase of UPEC (Anderson et al., 2003; Kim, 2003, 2008). Ag43 is also involved in the adhesion of NMEC to HBMECs (Ulett et al., 2007). The ATs UpaB and UpaC and their homologs are found in UPEC and in various strains of *E. coli* (Allsopp et al., 2012). UpaC promotes adhesion to abiotic surfaces and biofilm formation, while UpaB promotes adhesion to components of the extracellular matrix. *In vivo* experiments have suggested that UpaB, unlike UpaC, is important for bacterial fitness during UTI. Finally, the UpaG autotransporter promotes UPEC biofilm formation on abiotic surfaces and facilitates binding to extracellular matrix proteins, fibronectin, and laminin (Valle et al., 2008).

Extraintestinal *E. coli* PAIs also produce serine protease ATs that are designated as SPATEs (serine protease autotransporters of Enterobacteriaceae) such as Tsh (temperature sensitive hemagglutinin), Hbp (hemoglobin binding protein or hemoglobin protease), Sat (secreted autotransporter toxin), Vat (vacuolating autotransporter toxin) and Pic (protein involved in colonization, cf. DEC section). Tsh was initially identified in APEC as a mannose-resistant hemagglutinin that is overexpressed at low temperatures (Provence and Curtiss, 1994). It is observed in more than half of APEC isolates and its prevalence increases in high-lethality isolates (Dozois et al., 2000). The association of Tsh with the virulence of APEC isolates was further reinforced by the detection of fewer and less pronounced lesions in the air sacs of chickens infected with a Tsh mutant than with the wild type strain (Dozois et al., 2000). The *tsh* gene is also observed in UPEC (4.5%) and NMEC (11.5%) (Ewers et al., 2007). Frequently encoded by plasmids, *tsh* is also observed in PAIs and promotes adhesion to red blood cells, hemoglobin, and the extracellular matrix proteins fibronectin and collagen IV (Kostakioti and Stathopoulos, 2004). Tsh only differs from Hbp by two amino acid residues in the passenger domain and probably shares functional similarities regarding the breakdown of hemoglobin factor V and mucin (Dautin, 2010; Tapader et al., 2019).

The *vat* gene was also originally identified in an APEC strain as a cytotoxin of chicken embryonic fibroblast cells that contributes to avian cellulitis infection (Parreira and Gyles, 2003). It only shares 78% identity with Tsh and has different proteolytic activities (Parham et al., 2005b). However, Tsh/Hbp, Vat and Pic have mucinolytic activity and enhance epithelium colonization (Dautin, 2010; Gibold et al., 2016; Tapader et al., 2019). Pic and Tsh/Hbp also target the major human leukocyte-adhesion molecules CD43, CD44, CD45, and CD93, thereby deregulating leukocyte migration and inflammation (Ruiz-Perez et al., 2011). The Sat ATs induce vacuolation of renal cells, and the serine-protease activity is required for the cytopathic effects of Sat (Guyer et al., 2002; Maroncle et al., 2006). Mention has been made of the role of Sat in breaking down the glomerular barrier to allow bacteria to pass into the blood (Nataro and Kaper, 1998). Three new SPATEs—Sha (Serine-protease hemagglutinin autotransporter), TagB and TagC (tandem autotransporter genes B and C)—have also been recently reported in an ExPEC O1:K1 strain isolated from turkeys and they induce cytopathic effects on a bladder epithelial cell line (Habouria et al., 2019). However, their functional role in UTIs remains to be investigated.

SslE/YghJ (secreted and surface associated lipoprotein) is secreted by the type II secretion system and probably anchored at the bacterial surface in ExPEC. It is a metalloprotease domain initially observed in NMEC (Tapader et al., 2019). SslE, which is widely found in both commensal and pathogenic *E. coli*, has greater prevalence in pathogenic isolates than in commensal isolates, which may also be defective for its secretion (Moriel et al., 2010). SsIE targets mucins and has cytotoxic and proinflammatory activities that can promote the virulence of pathogenic *E. coli* and sepsis in neonates (Nesta et al., 2014).

#### Iron Uptake Systems Encoded by PAIs

Iron is essential for bacteria because of its involvement in many metabolic functions, such as the transport of oxygen and electrons. Most iron in humans is complexed with the transport molecules transferrin (found in blood) and lactoferrin (found in the digestive tract and in salivary and pulmonary secretions) and with reserve molecules (ferritin) or it is incorporated into the heme of hemoglobin and myoglobin. This complexation results in iron limitation, which in turn serves as one of the innate defenses against the survival of bacteria within hosts. To use iron from the host, bacteria have developed capture systems that target these iron-complexed forms within citrate ions (fec system) or heme (chu system). The bacteria use transport systems (Sit systems) or produce PK-NRP compounds called siderophores that capture iron from host transporters or reserve systems and transport it back to the bacteria via specific receptors (Braun, 2003). The siderophore enterobactin, which is found in almost all *E. coli*, is encoded by the core genome. Thus, Lipocalin-2, which is capable of sequestering enterobactin, prevents its uptake by the bacteria via the FepA receptor (Fischbach et al., 2006). Therefore, ExPEC acquires PAI-encoding iron-uptake systems to thwart this host defense mechanism. The most frequently encountered siderophores are aerobactin, yersiniabactin and salmochelin (Braun, 2003). Salmochelin is a modified enterobactin formed by glycosylation of one of its derivatives, and this modification makes salmochelin insensitive to inhibition by human Lipocalin-2 (Fischbach et al., 2006). The PAI-encoded siderophores salmochelin and yersiniabactin are both produced at significantly higher levels in ExPEC isolated from urine than in rectal isolates and could play a greater role than enterobactin within the human urinary tract (Henderson et al., 2009). In a newborn rat meningitis model, salmochelin plays an important role in maintaining a high level of bacteremia, which is a necessary step for bacterial crossing of the blood-brain barrier (Nègre et al., 2004).

Receptor-mediated uptake of heme is another iron-uptake mechanism encoded by ExPEC PAIs. Two outer membrane heme receptors, ChuA and Hma, have been characterized, and both were required for bacterial optimal fitness in a UTI mouse model (Hagan and Mobley, 2009). ChuA could also be important during IBC formation (Reigstad et al., 2007). The PAI-encoded Sit system transports manganese and ferrous iron into the ExPEC cytoplasm and enhances resistance to oxidative stress. The *sit* genes are upregulated during murine UTI and are expressed during human UTIs, suggesting that they contribute to urofitness (Snyder et al., 2004; Hagan et al., 2010).

#### Protectins Encoded by PAIs

Protectins are the bacterial elements that protect bacteria against host weapons, such as antibacterial factors (i.e., defensins, D-serine), serum complement and immune cells. PAI-encoded capsules (K antigen) confer this type of protection (Whitfield, 2006). The resulting homopolymers exposed at the surface of the bacteria can resemble various glycoconjugates found within vertebrate hosts (e.g., sialic acid), such as K1 or K4 capsules, and can contribute to the bacterium’s immune-evasion strategy. The K1 antigen is associated with *E. coli* strains responsible for invasive infections (Sarff et al., 1975). K1 antigen is critical for the induction of a high degree of bacteremia, is associated with a high resistance to both phagocytosis and serum and is required for bacterial crossing of the blood-meningeal barrier (Opal et al., 1982; Cross et al., 1984; Kim et al., 1992). Other PAI-encoded protective elements are surface proteins such as the Iss (increased serum survival) protein, which has been implicated in serum resistance (Xu et al., 2018).

D-serine is a bacteriostatic amino acid that is found in urine and brain (Cosloy and McFall, 1973; Kumashiro et al., 1995). UPEC and NMEC strains frequently harbor a PAI-encoded *dsdCXA* locus (Bloom and McFall, 1975; Moritz and Welch, 2006). The *dsdCXA* locus has been hypothesized to be important for UPEC/NMEC pathogenesis. Accordingly, isogenic strains defective in *dsdA* have a growth defect in urine relative to *dsdA* wild-type strains. However, *dsdA* neither positively nor negatively affected the ability of UPEC strain CFT073 to colonize urinary tract in a mouse model (Hryckowian et al., 2015).

## Diarrheagenic *Escherichia coli*

As already described above, InPEC essentially regroups (i) *E. coli* strains responsible for diarrhea (DEC) and (ii) AIEC, which are not involved in diarrhea but are involved in inflammatory bowel diseases. Among the DEC, the EPEC and EHEC have undoubtedly received the most attention regarding PAIs. By contrast, little information is available about PAIs in DAEC, except for the presence PAI *_*pic/set*_* in some strains (Czeczulin et al., 1999). Compared to ExPEC, the information about PAIs in DEC is generally scarce.

### Physiopathology of DEC

The six recognized categories of DEC are primarily based on clinical symptoms, including the type of diarrhea, related syndromes or bacterial interaction with intestinal epithelium cells, as revealed by histological observation, as well as associated molecular determinants of virulence and pathogenicity (Nataro and Kaper, 1998; Kaper et al., 2004).

ETEC are mainly associated with traveler’s diarrhea but can also appear in weanling diarrhea among children (Nataro and Kaper, 1998). The diarrhea is generally not bloody, but watery, and is sometimes associated with vomiting that ranges from mild to severe purging, similar to cholera. The pathogenicity of ETEC mainly relies on heat-labile (LT) and/or heat-stable (ST) enterotoxins responsible for net secretion of intestinal fluid (Turner et al., 2006a; Sack, 2011). The LT form belongs to the cholera toxin family and is secreted by a Type II secretion system (T2SS) with the contribution of LeoA (labile enterotoxin output) for its efficient secretion (Tauschek et al., 2002; Jobling and Holmes, 2006). Unlike the cholera toxins that are secreted as free soluble proteins, LT associates with the outer membrane vesicles by binding to LPS (Horstman and Kuehn, 2002). Sequence divergences confer LT with the ability to bind different ganglioside receptors that mediate entry into the host cell (Fukuta et al., 1988; Jobling and Holmes, 2006). LT is an ADP-ribosylating toxin that inactivates the GTPase activity of the G protein complex by ADP-ribosylation. This, in turn, leads to prolonged activation of the adenylate cyclase activity and elevated intracellular levels of cAMP and results in electrolyte release and stimulation of intestinal excretion (Spangler, 1992; Kaper et al., 2004; Jobling and Holmes, 2006).

By contrast, ST can be discriminated into type I (ST-I, also called STa) and type II (ST-II, also called STb), which target different receptors (Peterson and Whipp, 1995; Turner et al., 2006a). ST-I binds and activates a guanylate cyclase C receptor and the subsequent increase in intracellular cGMP levels activates the cystic fibrosis transmembrane conductance regulator (CFTR) chloride channel (Vaandrager, 2002; Turner et al., 2006a). ST-II, which binds the acidic glycosphingolipid sulphatide, activates a pertussis toxin-sensitive GTP-binding regulatory protein that ultimately activates CFTR (Peterson and Whipp, 1995; Fujii et al., 1997; Rousset et al., 1998). Surface colonization is an important in step in the physiopathology of ETEC, which involves a large and diverse repertoire of pili, called colonization factor antigens (CFAs), that bind to different receptors at the surface of the host cells (Turner et al., 2006a; Isidean et al., 2011; Ageorges et al., 2020). Additional virulence factors can be present, including some surface-exposed adhesins like TibA or Tia, the toxins ClyA and EAST1, or the protease EatA; however, this does not appear to be a common feature among ETEC (Turner et al., 2006a,b).

Enteropathogenic *E. coli* infection manifests as acute diarrhea in infants and is often associated with vomiting (Nataro and Kaper, 1998; Kaper et al., 2004). A hallmark of EPEC is its attaching and effacing (A/E) histopathology. This is accompanied by intimate attachment of bacterial cells expressing cell-surface intimin (Eae) with the cognate translocated intimin receptor (Tir) expressed on the surface of eukaryotic cell following injection into their cytosol by a Type III, subtype a, secretion system (T3aSS) (Nougayrède et al., 2003; Desvaux et al., 2006; Lai et al., 2013). A large repertoire of T3aSS effectors participate in the infection process (Dean et al., 2005; Dean and Kenny, 2009). However, and despite decades of investigations, the exact molecular mechanisms by which EPEC induce diarrhea are not well understood (Ochoa and Contreras, 2011).

The presence or absence of plasmid pEAF (*E. coli* adhesion factor) encoding bundle-forming pili (BFP), can be used to further categorize EPEC into typical (tEPEC) and atypical EPEC (aEPEC), respectively (Trabulsi et al., 2002; Hernandes et al., 2009; Hu and Torres, 2015). In tEPEC, BFP are type 4 pili (T4P) encoded by the *bfp* operon and induce a localized adhesion (LA) pattern at the surface of intestinal epithelial cells to form microcolonies (Scaletsky et al., 1984; Ageorges et al., 2020). In aEPEC, no localized LA patterns are observed but instead localized-adhesion-like (LAL) or aggregative adhesion (AA) patterns form loose bacterial cell clusters involving alternative adhesion factors such as EspA filaments, LifA (lymphocyte inhibitory factor) or ECP (E. coli common pili) (Rodrigues et al., 1996; Trabulsi et al., 2002; Ageorges et al., 2020).

EHEC are responsible for pediatric diseases ranging from watery diarrhea to hemorrhagic colitis that can further lead to thrombotic microangiopathies, especially hemolytic and uremic syndrome (HUS) and thrombotic thrombocytopenic purpura (TTP) (Karmali et al., 1983; Ruggenenti and Remuzzi, 1998; Tarr et al., 2005; Tarr, 2009; Wada et al., 2014). EHEC are primarily characterized by the expression of the key virulence shigatoxin (Stx) that binds to glycosphingolipid Gb3 before penetrating the cell and inducing apoptosis of kidney epithelial cells (Melton-Celsa, 2014). Nonetheless, and somewhat surprisingly, the way that Stx crosses the intestinal mucosa is not fully elucidated. While tEHEC possess the locus of enterocyte effacement (LEE) as in any EPEC, the aEHEC do not express the injectisome and instead rely on alternative monomeric and multimeric surface colonization factors, such as adhesins and pili (McWilliams and Torres, 2014; Monteiro et al., 2016; Ageorges et al., 2020). Similar to EPEC, however, the precise molecular mechanisms that induce bloody diarrhea remain elusive (Croxen et al., 2013; Stevens and Frankel, 2014).

Of note, and contrary to EPEC, A/E lesions are never detected on colonic biopsies from EHEC infection (Nataro and Kaper, 1998), although they are observed *in vitro* using epithelial cell culture or human colonic explants (Golan et al., 2011; Battle et al., 2014; Lewis et al., 2015). Some *E. coli* isolated from other sources (environment, animals, food and the agri-food chain) can be genotypically characterized as possessing the *stx* gene and can be regrouped as shigatoxin-encoding *E. coli* (STEC, or shigatoxin-producing *E. coli* when Stx production has been experimentally confirmed). The pathogenicity of EHEC has been clinically ascertained, and STEC can have very different virulence levels, up to hyper-virulence; alternatively, they can even be potentially avirulent and are not systematically and rigorously considered as pathogenic (Karmali et al., 2003; Caprioli et al., 2005; Laing et al., 2009; Monteiro et al., 2016). With more than 400 distinct serotypes revealed in STEC, only a handful of EHEC serotypes has been clinically associated with epidemic outbreaks, although they can all be potentially involved in human infections (Karmali et al., 2003; Blanco et al., 2004; Cristancho et al., 2008; Mathusa et al., 2010). The search for genetic markers has been the Grail for decades; nevertheless, the differences in the virulence level is most certainly associated with various compensatory combinations of molecular determinants, including PAIs, together with regulatory networks that can act at various levels and not just regarding the transcriptional aspects of genetic expression (Ageorges et al., 2020).

After ETEC, EAEC is the second most common cause of tourista that causes acute and sometimes persistent diarrhea (Jenkins, 2018). The diarrhea is typically non-bloody and without inflammation or emetic symptoms. Upon adhesion onto intestinal epithelial cells, EAEC exhibit a typical stacked brick-like pattern known as aggregative adhesion (Kaper et al., 2004). EAEC form a loose biofilm at the mucosal surface involving pili and especially AAF (aggregative adhesion fimbriae, encoded by a large plasmid pAA) (Kaper et al., 2004; Ageorges et al., 2020). Additional virulence factors include the pAA-encoded enterotoxins EAST1 and Pet (plasmid-encoded toxin) and the chromosome-encoded ShET1 (Shigella enterotoxin 1) and Pic (protein involved in intestinal colonization) (Chaudhuri et al., 2010; Jenkins, 2018). However, the molecular basis that mediates diarrhea upon infection by EAEC remains to be elucidated.

Extensive and compelling evidence indicates that EIEC and *Shigella* cannot be discriminated from a taxonomic point of view as they both belong to the same species, *E. coli*, and basically correspond to the same pathotype (Lan et al., 2004; Peng et al., 2009; Chaudhuri and Henderson, 2012; Pettengill et al., 2015; Michelacci et al., 2016). Differences in the virulence level have sometimes been put forward, with *Shigella* considered hyper-virulent relative to EIEC, which generally induces less severe disease. Nevertheless, significant exceptions do exist and the intestinal illness is in fact indistinguishable (Parsot, 2005; Michelacci et al., 2016; Belotserkovsky and Sansonetti, 2018; Hendriks et al., 2020). As with EHEC/STEC, the search for genetic markers that could solely explain the difference in the virulence levels might have somehow hindered the potential in the heterogeneity of the regulation of the genetic expression from one EIEC/*Shigella* strain to another as well as between individual cells in an isogenic population. The hallmarks of EIEC/*Shigella* are the invasion plasmid pINV, which encodes a T3aSS (like in EPEC and EHEC), and cognate effectors of key importance in host invasion of this intracellular pathogen, which have been extensively investigated (Zagaglia et al., 1991; Lan et al., 2001, 2003; Lan and Reeves, 2002; Schnupf and Sansonetti, 2019). Additional virulence factors include the mucinase Pic (previously called ShMu for Shigella haemagglutin and mucinase), also found in EAEC and contributing to the initial step of intestinal colonization (Henderson et al., 1999; Parham et al., 2004). Another is the protease SigA (*Shigella* immunoglobulin A protease) that contributes to the watery prodromal phase of the infection (Al-Hasani et al., 2000; Chua et al., 2015), together with the enterotoxins ShET1 and ShET2 (Henderson et al., 1999). The exact function and mode of action of the latter enterotoxins remain to be determined (Mattock and Blocker, 2017; Belotserkovsky and Sansonetti, 2018).

DAEC, rather than DEC, are essentially involved in diarrhea in young children (Servin, 2005, 2014), and some DAEC are ExPEC involved in urinary tract infections. EIEC are known to preferentially colonize the colon, as well as the distal ileum for EHEC, whereas the ETEC and EPEC colonize the small intestine, as well as the colon for EAEC (Clements et al., 2012). By contrast, the primary site of intestinal colonization of DAEC has not been determined (Servin, 2014). DAEC exhibits a scattered adhesion pattern on intestinal epithelial cells, called diffuse adhesion (Kaper et al., 2004; Croxen et al., 2013). DAEC have previously been subdivided into those expressing and those not expressing Afa/Dr (afimbrial adhesin/decay-accelerating factor receptor) adhesins (Servin, 2005), but the latter subclass is now considered to belong to aEPEC, leaving Afa/Dr adhesins as a hallmark of DAEC (Servin, 2014). Previously, *daaE* and/or *daaC* were briefly suggested as biomarkers of DAEC, but F1845 pili are rare among DAEC strains (Campos et al., 1999). The group of Afa/Dr adhesins is quite diverse and can induce rearrangement of the cytoskeleton upon binding to the eukaryotic cell and can even promote bacterial internalization (Servin, 2005; Le Bouguénec and Servin, 2006). Additional virulence factors include *pks* island products (such as colibactin), flagella and the secreted autotransporter toxin (Sat) (Servin, 2014). Notably, while some DAEC strains have been sequenced (Lindsey et al., 2018; Tang et al., 2019), no complete assembled genomes are as yet available. In addition, contrary to EHEC, ETEC, ETEC or EAEC with E. coli strains EDL933, H10407, E2348/69 or O42, respectively (Iguchi et al., 2009; Chaudhuri et al., 2010; Crossman et al., 2010; Latif et al., 2014), no prototypical DAEC strains have been sequenced to date, which limits our understanding of PAIs within this DEC pathotype.

### PAI-Encoded Virulence Factors of DEC

**FIGURE 4:**
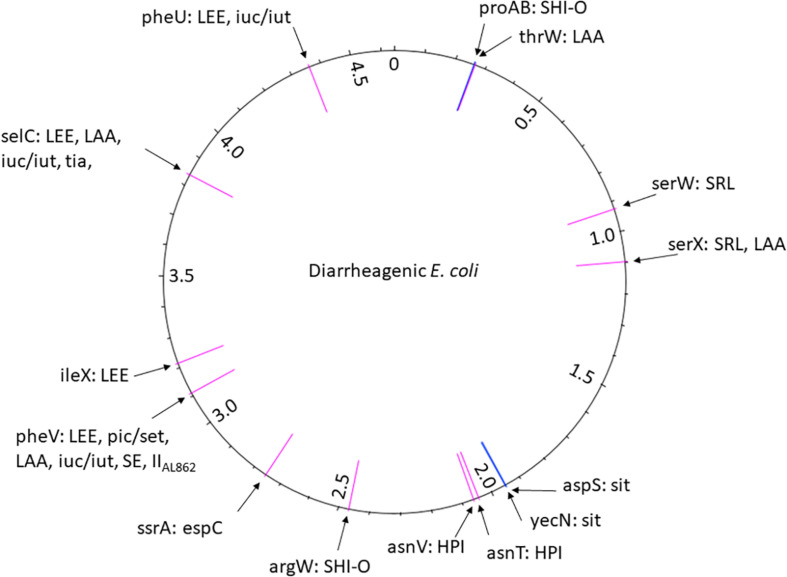
The position of PAI insertion sites in the diarrheagenic *E. coli* pathotypes. The name of the genetic features associated with the insertion is indicated with the name of PAIs found at the corresponding site. The position of the insertion site is indicated in pink for tRNA genes and in blue for coding genes. The numbers reported in the inner ring are the positions (megabase) in the genome of the non-pathogenic *E. coli* strain MG1565 used as a reference.

While PAIs were originally described in ExPEC, they later appeared to be present in InPEC. To date, 13 PAIs have been characterized, some of which are present in different DEC pathotypes (Figure 4; Table 3).

**TABLE 3:** Features of PAIs found in the different DEC pathotypes.

Name	Alternative names	Insertion site	Size (kb)	Main determinants encoded within the PAI^*a*^	DEC^*b*^
LEE PAI		*selC, pheV, pheU*	35.6-111	T3aSS apparatus, Intimin, Tir, Ter system*, LifA*	EPEC, tEHEC
PAI *_*pic/set*_*	SHI-1, She, PAI II_*CFT*__073_	*pheV*	46.6-71.6	Pic, ShET1	EAEC, EIEC, EHEC, EPEC, DAEC
LAA PAI		*pheV, selC, ileX, serX, thrW*	86.3	SisA, Hes, IhA, LesP, PagC, SlhA, Ag43	aEHEC, EPEC, EAEC
PAI *_*iuc/iut*_*	SHI-2, SHI-3	*selC, pheU, pheV*	21-30	Aerobactin, ShiA*, ShiD*	EIEC
PAI *_*tia*_*		*selC*	46	Tia, LeoA	ETEC, EPEC, EHEC
PAI *_*tibA*_*		*–*	21.6	TibA	ETEC
SE PAI		*pheV*	8	SubAB, Tia, ShiA	aEHEC
SHI-O PAI		*proAB, thrW, argW*	4.6	GtrABV	EIEC, EAEC
SRL PAI		*serX, serW, orf58*	66	Strep^*R*^, Amp^*R*^, Cm^*R*^, Tet^*R*^, FecABCDE-I-R, AspR-DcuA	EIEC, EHEC
PAI *_*espC*_*		*orf360, ssrA*	15.2	EspC, VirA	EPEC, EHEC
PAI *_*sit*_*		*aspS, yecN*	10-34	SitABCD	EIEC,
HPI		*asnT, asnV*	40	Ybt	EAEC, EIEC, EPEC, ETEC, EHEC
PAI II_*AL*__862_		*pheV*	61	Afa/Dr, DeoK	DEC

*^*a*^Main virulence or niche factors. ^∗^Molecular determinants that can be absent from some version of the PAI. ^*b*^DEC pathotypes where the PAI can be present. EPEC, enteropathogenic *E. coli*, EHEC, enterohemorrhagic *E. coli*, tEHEC, typical EHEC (LEE positive); aEHEC, atypical EHEC (LEE negative); ETEC, enterotoxigenic *E. coli*; EAEC, enteroaggregative *E. coli*; EIEC, enteroinvasive *E. coli* (including *Shigella*); DAEC, diffusely adherent *E. coli*; DEC, diarrheagenic *E. coli* (pathotype undefined among the above).*

#### LEE PAI

The LEE is undoubtedly the most investigated PAI in DEC, and readers are referred to recent reviews solely dedicated to this PAI for detailed information (Kirsch et al., 2004; Parham et al., 2005a; Schmidt, 2010; Stevens and Frankel, 2014; Connolly et al., 2015; Franzin and Sircili, 2015; Kendall, 2016; Furniss and Clements, 2018; Turner et al., 2019). The presence of the LEE is the hallmark of EPEC, but it is also found in tEHEC (McDaniel et al., 1995; McDaniel and Kaper, 1997). As first described in prototypical EPEC strain E2348/69 (Perna et al., 1998; Iguchi et al., 2009) and EHEC strain EDL933 (or strain Sakai) (Hayashi et al., 2001; Perna et al., 2001; Latif et al., 2014), the core region of the PAI consists of 41 CDS, organized into (i) 5 operons named LEE-1 to LEE-5 encoding transcriptional regulators, including the master LEE-encoded regulator (Ler), chaperones and structural components of the T3aSS, as well as some effectors, (ii) one *grlAB* bicistron encoding transcriptional regulators, and (iii) some monocistrons. The T3aSS constitutes a major virulence factor that allows the assembly of a cell-surface needle structure, the injectisome which enables the direct transport of virulence effectors into a host cell (Cornelis, 2006; Desvaux et al., 2006; Brutinel and Yahr, 2008; Mueller et al., 2008; Diepold and Wagner, 2014, 2014; Platenkamp and Mellies, 2018; Lara-Tejero and Galán, 2019; Ageorges et al., 2020). The regulation and function of the T3aSS has been extensively reviewed and readers are invited to consult the above reviews for further details on this vast field of research. Apart from the LEE core region, a certain degree of variability exists between LEEs found in different EPEC or EHEC strains, with estimated sizes ranging from approximately 35 to 110 kb (Sperandio et al., 1998; Jores et al., 2005; Müller et al., 2009). The LEE core region can be completed by prophage genes, as in EHEC ELD933 or Sakai, whereas the fusion with OI-43/48 SpLE1 (Sakai prophage-like element 1) containing a *ter* operon conferring resistance to tellurite (Tarr et al., 2000; Hayashi et al., 2001). The OI-122 SplE3, which contains a *lifA* (*efa1*) region that encodes the virulence factor lymphostatin (Klapproth et al., 2000; Badea et al., 2003; Morabito et al., 2003) or some truncated versions of these islands, can also be found in some variants of the LEE PAI (Gärtner and Schmidt, 2004; Kirsch et al., 2004; Müller et al., 2009). The LEE PAI is inserted at the *selC* tRNA gene, but the *pheV* and *pheU* tRNA genes were also reported as alternative insertion sites for the LEE among these variants (Rumer et al., 2003; Bertin et al., 2004). Phylogenetic analyses based on selected genes indicated that the LEE core region could be discriminated into two main core clusters, one harboring *eae*-α and -γ and the other one harboring *eae*-β and -ε independently from the tRNA insertion site (Jores et al., 2004). Nonetheless, the current knowledge on the distribution of the LEE indicates complex events of genetic acquisitions and recombinational rearrangements in *E. coli* genomes and the evolutionary network remains quite incomplete (Jores et al., 2004; Rasko et al., 2008; Montero et al., 2019).

#### PAI *_pic/set_*

PAI *_*pic/set*_* is inserted into the *pheV* gene and was identified in EAEC, as well as in EIEC/*Shigella* (called SHI-1 PAI, previously also known as She) and UPEC (called PAI II_*CFT*__073_), and in some EHEC and EPEC strains; however, they differ in size, organization, gene composition and genome localization (Al-Hasani et al., 2001a,b; Behrens et al., 2002; Parham et al., 2005a). Island probing revealed this PAI to be quite unstable and to undergo spontaneous excision from the chromosome at a frequency of 10^–5^–10^–6^ (Rajakumar et al., 1997). The number of IS-like element flanking the gene (especially IS629 and IS911 also found around other genes encoding SPATEs) suggested that *pic* (previously called *she*) is part of a mobile element that could move independently of the PAI (Henderson et al., 1998, 1999; Parham et al., 2005a).

The *setB* and *setA* genes, contained within the *pic* gene but on the complementary strand, encode the oligomeric enterotoxin ShET1 (Fasano et al., 1995; Henderson et al., 1999; Behrens et al., 2002). The transcription of *setB* was induced in simulated human intestinal microbial systems, although the exact signals promoting gene expression remain to be identified (Behrens et al., 2002). The genetic expression of the *pic/set* locus is quite unusual and complex. At least three promoters, P*_*pic*_*1, 2, and 3, could direct the transcription of *pic* but with different forces and constitutive/inducible expression (Behrens et al., 2002). A Ps*_*etA*_* promoter was identified just upstream of setA, whereas a P*_*setB*_* was identified 1.5 kb upstream of *setB* with a silencer DRE (downstream regulatory element) region in between that repressed the expression of *setBA* without any effect on *pic* transcription (Behrens et al., 2002). Apart from regulation by its own promoter, *setA* also appears to be expressed by transcription readthrough from *setB*.

#### LAA PAI

The locus of adhesion and autoaggregation (LAA) is a composite 86-kb PAI composed of four modules (Montero et al., 2017). In addition to the host immune response suppressor SisA (shiA-like inflammation suppressor genes A) (Lloyd et al., 2009b), module I encodes an autoaggregative factor with Hes (hemagglutinin from STEC), a member of the Hra (heat-resistant agglutinin) family (Montero et al., 2014), together with module IV encoding Ag43, a member of the SAAT (self-associating autotransporter). Module III encodes the adhesin Iha (iron regulated gene homolog adhesin) and the SPATE LesP (LAA encoded SPATE), while module IV actually corresponds to the PAI previously known as PAI I_*CL*__3_ (Shen et al., 2004).

PAI I_*CL*__3_ was originally described is a hybrid genomic region that entered B1 *E. coli* genomes on multiple and independent occasions (Shen et al., 2004). It is composed of segments of the EHEC EDL933 OI-48 at both its extremities and two genomic segments, GS-I and GS-II. GS-I comprises fragments of the *Z1640* gene encoding the ShlB transporter family of the T2bSS, whereas GS-II is a composite region encoding a member of the ShlA exoprotein family secreted by the T5bSS. GS-II encodes several transposase genes, genes originating from EHEC EDL933 OI-122, including *pagC* (*phoP*-activated gene C), and an additional fragment of *Z1640* (Shen et al., 2004; Girardeau et al., 2009). Different variants and often deleted versions of PAI I_*CL*__3_ were reported as carried by genomic islands, including GI*pheV*-CR_*ICC*__168_ from *Citrobacter rodentium*, inserted at *pheV*, *selC* or *serW* tRNA (Girardeau et al., 2009). However, the mosaic structure of LAA PAI was not understood at the time, and these GIs represent complete and incomplete LAA PAI variants (i.e., comprising less or all of the four modules) belonging to two major lineages (Montero et al., 2017). In agreement with PAI I_*CL*__3_ investigations (Girardeau et al., 2009; Hauser et al., 2013), the LAA PAI appeared to be exclusively present in aEHEC in the first instance and could thus complement the absence of the LEE PAI (Colello et al., 2018, 2019).

#### PAI *_*iuc/iut*_*

The SHI-2 (Shigella pathogenicity island 2) PAI is inserted at the *selC* tRNA gene and encodes an integrase almost identical to its ortholog in the LEE PAI. It possesses an abundant number of partial transposases and IS elements, which are remnants of the multiple stepwise assembly of the island (Moss et al., 1999; Vokes et al., 1999). SHI-2 codes the *iucABCD* operon for synthesis of aerobactin (Lawlor and Payne, 1984), a hydroxamate siderophore involved in iron uptake (Vokes et al., 1999; Braun, 2003), as well as the *iutA* gene encoding the outer membrane receptor for aerobactin complexed to iron (Crosa, 1989). In *Shigella boydi*, a 21-kb PAI harboring the *iucABCD-iutA* operon but inserted at the *pheU* tRNA gene and possessing LEE prophage genes absent from SHI-2, was called SHI-3 (Purdy and Payne, 2001; Nie et al., 2006). Nonetheless, SHI-3 remains very closely related to the 30-kb SHI-2 in *S. flexneri* 2a SA100 (Vokes et al., 1999) and 23.8-kb SHI-2 in *S. flexneri* 5a M90T (Moss et al., 1999), and it belongs to the same family of PAI.

The *iut/iuc* operon is highly conserved in SHI-2 and SHI-3 and can be inserted in at different loci, especially in paralogs to *phe* tRNA genes (at least *pheV*, *pheR* and *pheU*) (Al-Hasani et al., 2001a; Nie et al., 2006). The SHI-2/3 PAIs are regrouped under the designation of PAI *iuc/iut* to rationalize the naming in a way similar to that of PAI *_*pic/set*_*. The PAI *_*iuc/iut*_* would have been acquired horizontally, as suggested by its high degree of conservation with the aerobactin operon harbored on plasmid pColV in some *E. coli* strains (Moss et al., 1999). SHI-2 also harbors several genes encoding the predicted cytoplasmic proteins, including ShiA (a putative quinone reductase) that has been demonstrated to attenuate inflammation by abrogating the innate T-cell response (Moss et al., 1999; Ingersoll et al., 2003; Ingersoll and Zychlinsky, 2006). SHI-2 also encodes potential integral inner membrane proteins, including ShiF (a putative transporter of the major facilitator superfamily), a dispensable auxiliary factor involved in aerobactin export and/or synthesis (Moss et al., 1999; Genuini et al., 2019). Like *pic/set* in SHI-1, the *shiF* and *shiG* genes overlap and would be transcribed in reverse orientation on a complementary strand, although experimental investigations of genetic regulation and function of ShiG are still required (Moss et al., 1999).

Importantly, the *imm* (immunity) gene (*shiD*) appears responsible for immunity to different colicins, including colicin V (ColV) (Vokes et al., 1999). Colicins are proteins produced by *E. coli* that cause cell lysis of sensitive strains (Cascales et al., 2007). In some strains, PAI *_*iuc/iut*_* is inverted and is thus expressed from the complementary strand, but it is also differently localized and orientated with respect to the *oriC* (Nie et al., 2006), which could have consequences on the expression of the aerobactin genes (Schmid and Roth, 1987; Eisen et al., 2000; Kothapalli et al., 2005). Among the EIEC/Shigella, the size of PAI *_*iuc/iut*_* clearly differs, but further investigations are needed to determine their diversity and to gain insight into their gene content, organization, phylogeny and distribution in *E. coli* genomes (Moss et al., 1999; Vokes et al., 1999; Yang et al., 2005).

#### PAI *^*tia*^*

The prototyptical ETEC strain H10407 contains 25 identified ROD (regions of difference) on the chromosome, two of which are currently viewed as PAIs with the *tia* (toxigenic invasion locus A) and *tibA* (toxigenic invasion locus b) loci (Elsinghorst and Weitz, 1994; Crossman et al., 2010). Tia is encoded in a large PAI of approximately 46 kb (corresponding to ROD-20), inserted at *selC* and flanked by direct repeat sequences (DRSs) identical to those found in PAI-1 in UPEC (Fleckenstein et al., 1996, 2000; Crossman et al., 2010). Tia can act both as an invasin, contributing to the invasion of intestinal epithelium cells (Bondì et al., 2017), and as an adhesin, binding to heparin-sulfate proteoglycans (Mammarappallil and Elsinghorst, 2000; Fleckenstein et al., 2002; Turner et al., 2006b). In addition to *int*, PAI *_*tia*_* encodes LeoA, an accessory factor for efficient LT secretion, as well as three additional CDS encoding a putative transport system (Fleckenstein et al., 2000). DNA hybridization studies indicate that PAI *_*tia*_* is present in numerous ETECs, as well as in some EPEC and EHEC (Fleckenstein et al., 2000). Nonetheless, the hybridization and multiplex PCR patterns exhibit a considerable level of heterogeneity, suggesting differences in the organization and content of the PAI. The spread of this PAI among DEC and broadly among *E. coli* strains requires further in-depth investigations (Swenson et al., 1996; Fleckenstein et al., 2000).

#### PAI *_*tibA*_*

In ETECs, TibA is encoded on a 21.6-kb PAI of low G + C content, corresponding to ROD-13 (Elsinghorst and Weitz, 1994; Crossman et al., 2010). Besides the *tibDBCA* operon, the PAI *_*tibA*_* codes 14 transposases and IS66, but the insertion site remain elusive. TibA belongs to the SAAT family, is secreted by the T5aSS and is glycosylated by the heptosyltransferase TibC (Masuda et al., 1983; Turner et al., 2006b). Besides cell aggregation and biofilm formation, this glycosylated surface protein is involved in adhesion to intestinal epithelial cells (Lindenthal and Elsinghorst, 1999). The functions of TibD and TibB are yet to be determined. The prevalence and distribution of PAI *_*tibA*_* among *E. coli* remains to be investigated, though it is not systematically present in all ETEC strains (Elsinghorst and Weitz, 1994; Crossman et al., 2010).

#### SE PAI

LEE-negative EHEC (i.e., aEHEC) contain an 8-kb PAI coding a *subAB*_2_ operon, expressing a subtilase (Michelacci et al., 2013). The *subAB*_1_ allelic variant is located on a plasmid, but no additional DNA regions corresponding to the PAI were identified, suggesting that it was not horizontally acquired from this plasmid. SubAB is a well-known cytotoxin inducing apoptosis in LEE-negative EHEC (Wang et al., 2007; Wolfson et al., 2008; Yahiro et al., 2012). This so-called SE (subtilase encoding) PAI also harbors genes encoding an integrase and a sulfate, as well as Tia and ShiA, and is inserted in the *pheV* tRNA gene. The presence of *tia*, as in PAI *_*tia*_*, and *shiA*, as in PAI *_*pic/set*_*, as well as the presence of *subAB*_2_ in some ETEC strains (Michelacci et al., 2013), raises questions about the origin and evolution of the SE PAI with respect to these PAIs and demands further phylogenetic analyses. The SE and LEE PAIs have the same insertion site, which led to the hypothesis that mutual exclusion events could have occurred between these PAIs competing for integration at *pheV* tRNA gene and could further explain the strong association of the subAB2 operon with aEHEC versus tEHEC (Orden et al., 2011; Michelacci et al., 2013). As with PAI *_*iuc/iut*_*, however, insertion at homologous *phe* tRNA genes cannot be excluded. In general, the prevalence of SE PAI in DEC, and in other *E. coli*, requires further in-depth investigations.

#### SHI-O PAI

The SHI-O PAI harbors genes involved in serotype conversion in *Shigella* by modification of the structure of the O-antigens (Allison and Verma, 2000; Ingersoll et al., 2002; Torres, 2004). SHI-O would appear to be a remnant of an ancient lysogenic bacteriophage that lost its ability to enter into a lytic cycle and underwent excision upon deletions and mutations of sensitive key regions. This PAI codes three genes, *gtrA*, *gtrB* and *gtrV*, that express the glycosyltransferases that allow differential glycosylation of bacterial lipopolysaccharide (LPS) and thus antigenic conversion (Huan et al., 1997; Adams et al., 2001; Sun et al., 2013). SHI-O was also shown to be inverted and to differ in its composition between *Shigella* strains (Nie et al., 2006) and to have a different insertion in *E. coli* (Allison and Verma, 2000). Besides the generation of a large variety of shigella serotypes, the modification of O antigens by SHI-O contributes to the modulation of the virulence level through different abilities to evade the host immune response (Robbins et al., 1992; Hong and Payne, 1997; Van den Bosch et al., 1997). The prevalence and distribution of SHI-O in *E. coli* has not been questioned as yet, although it is present in prototypical EAEC O42 (Chaudhuri et al., 2010).

#### SRL PAI

The SRL (shigella resistance locus) PAI is a 66-kb element containing at least 59 CDS, especially a 16-kb cluster designated as SLR with genes conferring resistance to streptomycin (*aadA1*), ampicillin (*oxa-1*), chloramphenicol (*cat*) and tetracycline (*tetABCD-R*) (Rajakumar et al., 1996; Turner et al., 2001, 2003). The SRL PAI is a mobile element flanked by short 14-bp DRSs, which can excise from its insertion site at tRNA gene *serX* (Turner et al., 2001). The deletion rate was estimated at approximately 10^–5^ and led to antibiotic-sensitive variants. The *int* (integrase) gene was required for precision deletion of the island through site-specific recombination. Alternative insertion sites are strongly suspected, such as *serW*, paralogous to *serX*, and *orf58* (Turner et al., 2003). Besides antibiotic resistance, SRL PAI encodes a functional ferric dicitrate iron transport system (*fecABCDE-I-R*), which expands the battery of iron acquisition mechanisms in the bacterial strain (Luck et al., 2001). Functional genetic analysis revealed that SRL PAI encoded a functional aspartate racemase converting D- to L-aspartate (AspR) and a cognate transporter (DcuA) enabling catabolism of D-asparate as a sole carbon source (Henríquez et al., 2020). Catabolism of D-amino acids, such as D-aspartate, as a carbon source and/or detoxification of their toxic effects on protein biosynthesis could have an important role in the colonization of the human gastrointestinal tract, an aspect which undoubtedly requires further in-depth investigations. The aspR and *dcuA* homologs are present in some EHEC O104:H4 isolates (Henríquez et al., 2020), but the broad prevalence of SLR PAI in *E. coli* needs thorough investigation, although this PAI seems quite prevalent in multiresistant *Shigella* isolates (Turner et al., 2003).

#### PAI espC

EspC (*E. coli* secreted protein C) is a SPATE with enterotoxic activity that induces necrosis and apoptosis in epithelial cells (Mellies et al., 2001; Navarro-Garcia et al., 2014; Serapio-Palacios and Navarro-Garcia, 2016). EspC actually cleaves the cytoskeletal actin-associated protein α-fodrin, the focal adhesion protein paxillin and a focal adhesion kinase (FAK). In EPEC, EspC is encoded in a 15.2-kb PAI inserted between *orf360* and tRNA *ssrA* genes, with respect to the *E. coli* K12 chromosome (Mellies et al., 2001). Besides five putative transposases, PAI *_*espC*_* encodes an integrase and a homolog to the virulence effector VirA from *S. flexneri* that is involved in cellular invasion and intracellular spreading (Campbell-Valois et al., 2015; Agaisse, 2016), as well as additional CDS with low sequence similarities that require further analysis to ascertain their function. The low G + C content of this PAI suggests it was acquired horizontally. PAI *_*espC*_* seems present in other DEC, including some EPEC and EHEC, but additional studies are needed to characterize the possible diversity in the genetic organization, prevalence and distribution (Mellies et al., 2001).

#### PAI *_*sit*_*

Originally described in *S. flexneri*, the PAI *_*sit*_* primarily harbors a *sitABCD* locus encoding a ferrous iron and manganese uptake system belonging to the ABC transporter family, homologous to Sit (salmonella iron transport) (Kehres et al., 2002; Runyen-Janecky et al., 2006; Fisher et al., 2009). The PAI *_*sit*_* is found in some commensal and pathogenic *E. coli* at various chromosomic locations, with insertion sites that fall into four clusters (Fisher et al., 2009). The size and orientation of the island seem to differ considerably, but further studies are required to assess the degree of variation in the gene content of the PAI *_*sit*_* (Chen and Schneider, 2006; Fisher et al., 2009). This PAI has multiple chromosomic insertion sites and a large number of genetic rearrangements and deletion and insertion sequences that would occur through homologous recombination rather than site-specific mechanisms. Therefore, this PAI appears quite unstable (Fisher et al., 2009).

#### HPI

The HPI (high pathogenicity island) is a PAI originally identified in *Yersinia enterocolitica* (Schubert et al., 1998; Carniel, 2001). It later appeared widely disseminated in *Enterobacteriaceae*, including DEC, but essentially in EAEC, as it is rarely present in EIEC/Shigella, EPEC, ETEC or EHEC (Czeczulin et al., 1999; Karch et al., 1999; Schubert et al., 2000). The HPI encodes an iron uptake system based on the siderophore yersioniabactin (Ybt) (Schubert et al., 2004b), which was demonstrated to be functional in several EAEC strains and contributes to the virulence (Carniel, 2001; Hu et al., 2005). While *fyuA* (also called *psn*) and *ybtPQ* (also called irp6-7) encode an outer-membrane receptor and an inner-membrane transporter for iron uptake, respectively, *irp1-5* and *ybtS* (*irp9*) are involved in the biosynthesis of Ybt (Carniel, 2001). In *E. coli*, the HPI is approximately 40 kb and is generally inserted at the *asnT* tRNA gene (Buchrieser et al., 1998; Schubert et al., 2004b). However, it can also be inserted at the *asnV* tRNA gene harbored in place of IS600, an additional 34.4-kb fragment constituting a functional ICE encoding a T4SS (Schubert et al., 2004a). This element could constitute the progenitor and missing link that would explain the dissemination of HPI among *E. coli*.

#### PAI II_*AL*__862_

*Escherichia coli* AL862 was primarily isolated from a septic patient with a cancer, but the PAI containing the *afa-8* operon, and further called PAI II_*AL*__862_, appeared as a preferential insertion into the *pheV* gene in several human and bovine pathogenic *E. coli* strains (Lalioui and Le Bouguénec, 2001; Redford and Welch, 2002). The *afa-8* operon, encoding the Afa/Dr adhesin AfaEVIII (Lalioui et al., 1999; Lalioui and Le Bouguénec, 2001), is expressed in some *E. coli* strains isolated from animals with diarrhea, including calves, pigs, and poultry (Gérardin et al., 2000). PAI II_*AL*__862_ can be identified in some human intestinal *E. coli* isolates but evidence of its implication in diarrhea in humans remains to be demonstrated (Le Bouguénec et al., 2001; Girardeau et al., 2003). This PAI is supposedly present in some DAEC, but investigations are still required (Servin, 2014). Among the four sRNAs encoded in PAI II_*AL*__862_, the thermoregulated sRNA AfaR further regulates the expression of the AfaD-VIII subunit. PAI II_*AL*__862_ also includes the *deoK* operon, which confers the ability to use deoxyribose as a carbon source and would increase competitiveness with respect to host infectivity (Lalioui and Le Bouguénec, 2001).

## Conclusion

As reviewed here above, PAIs are widespread in *E. coli* and are major actors in the genome plasticity of this bacterium. The PAIs have shaped the genome of this bacterial species, which further explains the emergence of different pathotypes that result in different intestinal and extraintestinal diseases. The transfer of these GIs in a one-step acquisition of the entire functional determinants, thereby enabling bacterial evolution in quantum leaps, opens the bacterium to new ecological niches. The functional elements encoded by PAIs include pathogenicity effectors, but they also contain regulatory elements involved in crosstalk between PAIs and the genetic background. This crosstalk may be key factors in genome evolution, including PAIs, and probably result in a diversity of expression profiles. This requires more investigations to obtain the best understanding of *E. coli* virulence and the apparent redundancy of pathogenicity factors in ExPEC.

As exemplified by the LEE and LAA PAIs, these genomic islands are also quite plastic in terms of their insertion or deletion events, resulting in PAIs of different sizes in different strains but with a common evolutionary history. This review has further stressed that, in some cases, further investigations are still required to fill the existing knowledge gaps, for example, about the phylogenetic relationship between SE PA, PAI *_*tia*_*, and PAI *_*pic/set*_* (Michelacci et al., 2013), which may belong to the same family as was later identified for PAI I_*CL*__3_ and PAI LAA (Montero et al., 2017). However, the PAI nomenclature is quite erratic and requires a more rigorous formulization to avoid different naming of homologous genomic islands, such as SH-2 and SH-3, found in different strains, as was done for HPI. This is necessary to facilitate PAI tracing and our understanding of their phylogeny and evolution.

Additional PAIs are most certainly present in *E. coli*; examples are the genomic islands OI-57 (Coombes et al., 2008; Imamovic et al., 2010), OI-122 (Karmali et al., 2003; Wickham et al., 2006; Konczy et al., 2008), OI-43/48 (Taylor et al., 2002; Yin et al., 2009; Ju et al., 2013) and GI-*selC*_17584__/__1_ (Schmidt et al., 2001; Saile et al., 2018) in EHEC or some ROD in ETEC (Crossman et al., 2010). However, beyond a correlation of their association with outbreaks or severe diseases, experimental confirmation is still required to demonstrate their involvement and exact role in *E. coli* virulence and pathogenicity.

The evolution of the pathogenesis of *E. coli* constitutes a challenge for public health authorities in both developed and developing countries. This is especially evident given the burden of disease and the emergence of new epidemic strains, as dramatically exemplified by the hybrid diarrheagenic EHEC/EAEC O104:H4 strain (Navarro-Garcia, 2014; Prager et al., 2014; Kampmeier et al., 2018). The efficient horizontal gene transfer and high genetic plasticity of the *E. coli* genome, at both the plasmidic and chromosomic levels, represent potential public health issues that require a full comprehension and determination of the molecular mechanisms at play in the emergence of new *E. coli* pathotypes and/or *E. coli* strains with higher virulence levels. The description of numerous *E. coli* pathotypes with respect to intestinal and extraintestinal pathogenic diseases should not hide the fact that *E. coli* is primarily a normal and harmless commensal of the human intestinal microbiota, and that all these strains belong to the very same and unique bacterial species, *E. coli* (including *Shigella*).

To avoid biases in our understanding of *E. coli* physiology when solely focusing on anthropocentric medical and pathogenic aspects, we must consider not only pathogenic human and animal isolates but also the commensal strains that occur all along the food chains. Very diverse selection pressures can be encountered in the trophic network and can work together to drive evolution. In the various ecosystems that *E. coli* calls home (e.g., animal and human GIT, food matrices and soil, water, agri-food environments), the colibiote consists of different commensal and/or pathogenic *E. coli* strains co-existing and interacting along with the rest of the microbiota of the biocoenoses in the various environmental conditions of the biotopes. *E. coli* is not intentionally pathogenic, as suggested by its pathoadaptation (Maurelli, 2007; Rahman and Gadjeva, 2014; Pasqua et al., 2017). However, ecocentric and biocentric views allow the perception that these different levels of interactions can induce evolution and selection of genetic traits for specific niche factors for the necessity of fitness and adaptation, which can inadvertently, collaterally, coincidentally and/or randomly result in virulence as a by-product of commensalism (Le Gall et al., 2007; Tenaillon et al., 2010; Hill, 2012; Leimbach et al., 2013). Integrating the physiopathology to the ecophysiology of this species is certainly the next frontier in understanding the forces at play in the *E. coli* genome plasticity and the emergence of new virulence traits and/or pathotypes.

## Author Contributions

MD and RB wrote the first overall draft of the manuscript. RB drew the original pictures. MD, GD, RBe, NB, JD, and RBo wrote sections of the manuscript. RB contributed to conceptualize the overarching aims and had management as well as coordination responsibility for the execution of the work. All authors contributed to the critical revision of the manuscript, read and approved the submitted version.

## Conflict of Interest

The authors declare that the research was conducted in the absence of any commercial or financial relationships that could be construed as a potential conflict of interest.

## Abbreviations

A/E: attaching and effacing
AA: aggregative adherence
AAF: aggregative adherence fimbriae
ABU: asymptomatic bacteriuria
aEHEC: atypical enterohemorrhagic *E. coli*
aEPEC: atypical enteropathogenic *E. coli*
Afa: afimbrial adhesin
Ag43: antigen 43
AIEC: adherent-invasive *E. coli*
APEC: avian pathogenic *E. coli*
AT: autotransporter
CASPR1: contactin-associated protein 1
CDS: coding sequence
Cdt: cytolethal and distending toxin
CFA: colonization factor antigen
CFTR: cystic fibrosis transmembrane conductance regulator
CLB: colibactin
CNF-1: cytotoxic necrotizing factor-1
DAEC: diffusely adherent *E. coli*
DEC: diarrheagenic *E. coli*
Dr: decay-accelerating factor receptor
DRE: downstream regulatory element
DRS: direct repeat sequences
EAEC: enteroaggregative *E. coli*
ECP: *E. coli* common pili
EHEC: enterohemorrhagic *E. coli*
EIEC: enteroinvasive *E. coli*
EPEC: enteropathogenic *E. coli*
EspC: *E. coli* secreted protein C
ETEC: enterotoxigenic *E. coli*
ExPEC: extraintestinal *E. coli*
FAK: focal adhesion kinase
GbO3: globotriosylceramide
GbO4: globotetraosylceramide
GbO5: globopentaosylceramide
GI: genomic islands
GS: genome segment
HBMEC: human brain microvascular endothelial cell
Hbp: hemoglobin binding protein or hemoglobin protease
HPI: high-pathogenicity island
Hra: heat-resistant agglutinin
HT: horizontal transfer
HUS: syndrome hemolytic and uremic
IBC: intracellular bacterial community
IBD: inflammatory bowel disease
ICE: integrative and conjugative elements
Iha: iron regulated gene homolog adhesin
InPEC: intestinal pathogenic *E. coli*
LA: localized adherence
LAA: Locus of adhesion and autoaggregation
LAL: localized-adherence like
LEE: locus of enterocyte effacement
Ler: LEE-encoded regulator
LifA: lymphocyte inhibitory factor
LPS: lipopolysaccharide
LT: heat-labile enterotoxin
NlpI: new lipoprotein I
NMEC: neonatal meningitis *E. coli*
OmpA: outer membrane protein A
PagG: *PhoP*-activated gene C
PAI: pathogenicity islands
Pap: pyelonephritis associated pili
Pet: plasmid-encoded toxin
PF: Pathogenicity factors
Pic: protein involve in colonization
RDF: recombination directionality factor
RTX: repeat-in-toxin
SAAT: self-assocoiating autotransporter
Sat: secreted autotransporter toxin
SEPEC: sepsis-associated *E. coli*
Sha: serine-protease hemagglutinin autotransporter
ShET1: shigella enterotoxin 1
ShET2: shigella enterotoxin 2
SHI: shigella pathogenicity island
SigA: *Shigella* immunoglobulin A protease
Sit: Salmonella iron transport
SPATE: serine protease autotransporters of Enterobacteriaceae
SpLE: sakai prophage-like element
SslE: secreted and surface associated lipoprotein
ST: heat-stable enterotoxin
STEC: shigatoxin-producing *E. coli*
Stx: shigatoxin
T2SS: type II secretion system
T3aSS: type III, subtype a, secretion system
T3SS: type III secretion system
T4SS: type IV secretion system
Tag: tandem autotransporter gene
TcpC: TLR domain containing-protein C
tEHEC: typical enterohemorrhagic *E. coli*
tEPEC: typical enteropathogenic *E. coli*
Tia: toxigenic invasion locus A
TLR: toll-like receptor
Tsh: temperature sensitive hemagglutinin
TTP: thrombotic thrombocytopenic purpura
UPEC: uropathogenic *E. coli*
UTI: urinary tract infection
Vat: vacuolating autotransporter toxin
Ybt: yersioniabactin.
